# Do blast induced skull flexures result in axonal deformation?

**DOI:** 10.1371/journal.pone.0190881

**Published:** 2018-03-16

**Authors:** Harsha T. Garimella, Reuben H. Kraft, Andrzej J. Przekwas

**Affiliations:** 1 Department of Mechanical and Nuclear Engineering, The Pennsylvania State University, University Park, PA, United States of America; 2 Computational Medicine and Biology Division, CFD Research Corporation, Huntsville, AL, United States of America; Beihang University, CHINA

## Abstract

Subject-specific computer models (male and female) of the human head were used to investigate the possible axonal deformation resulting from the primary phase blast-induced skull flexures. The corresponding axonal tractography was explicitly incorporated into these finite element models using a recently developed technique based on the embedded finite element method. These models were subjected to extensive verification against experimental studies which examined their pressure and displacement response under a wide range of loading conditions. Once verified, a parametric study was developed to investigate the axonal deformation for a wide range of loading overpressures and directions as well as varying cerebrospinal fluid (CSF) material models. This study focuses on early times during a blast event, just as the shock transverses the skull (< 5 milliseconds). Corresponding boundary conditions were applied to eliminate the rotation effects and the resulting axonal deformation. A total of 138 simulations were developed– 128 simulations for studying the different loading scenarios and 10 simulations for studying the effects of CSF material model variance–leading to a total of 10,702 simulation core hours. Extreme strains and strain rates along each of the fiber tracts in each of these scenarios were documented and presented here. The results suggest that the blast-induced skull flexures result in strain rates as high as 150–378 s^-1^. These high-strain rates of the axonal fiber tracts, caused by flexural displacement of the skull, could lead to a rate dependent micro-structural axonal damage, as pointed by other researchers.

## Introduction

Blast-induced traumatic brain injury (bTBI) is a common type of battlefield injury. A report by Warden et al. [1] stated that 56% of the blast-exposed patients among the military personnel were diagnosed with moderate to severe TBI with the remainder considered to have mild TBI. Blast impact can be classified [2] based on the type of external loading–primary, secondary, tertiary and quaternary. Primary blast-induced injuries are a direct result of the exposure of the human head to overpressure shock waves. Potential injury mechanisms associated with primary blast injury are not fully understood and are still being investigated. These possible blast injury mechanisms include: 1) compression of thorax and resulting pressure surge to the human head [3,4], 2) axonal strains due to rotational accelerations experienced by the human head [5,6], 3) direct transmission of blast energy through cranium (transmission through thickness) [4], 4) injury due to wave transmission through skull flexures from skull to brain [7–9] and blast-induced cavitation that cause cellular damage [9].

An important aspect of primary blast injury, that is different from a blunt impact injury (say from sports or vehicular accidents), is the time scale of the loading. In a blast scenario, depending on the explosive charge and distance an individual is from the detonation point, the primary overpressure can propagate over the body in less than 5 ms. Once the shock wave meets the head, some of its energy is transferred into the brain, resulting in tension and compression waves traveling at the speed of sound (**≈**1500 m/s) leading to high intracranial pressures almost immediately after the loading. Shear waves, during an impact loading condition, travel at lower speeds (**≈**10 m/s) in the brain [10] leading to a slow evolution of the strains along the axons. Since blast loading occurs very fast and has a time span of 2–6 ms, the strain that the brain experiences in the short time (<10 ms) following the blast loading has been found to be small or of secondary interest.

However, there has been computational and experimental evidence that shows the strains can be significant [11] at a later time. Nevertheless, the loading time is short, and tissue strains are small. Therefore, previous studies have used pressure or stress response in the brain to quantify the injury [12,13]. However, the mechanical response (stress/pressure), obtained at a tissue level, might not be fully representative of the pathological changes (such as axonal swellings along the axonal tracts) in the brain. These changes might be more accurately captured by studying the mechanical response at an axonal fiber tract level [14]. Therefore, there is a need for the use of more physiologically relevant head models (with axonal fiber information) and injury criterion (such as axonal strain and strain rate) [15]. Besides this, with recent studies showing abnormalities in diffusion tensor imaging (consistent with white matter injury) of subjects exposed to primary blast wave [16–18], there is an increased importance of studying the evolution of these white matter disruptions under blast loading scenarios. These white matters disruptions might be quantified by calculating the axonal damage (calculated from axonal strain and strain rates) from the finite element models (developed with incorporated axonal fiber information) [19, 20]. Axonal strain, however, is a delayed effect as demonstrated by the human head simulations performed by Dagro et al. [11]. With the recently increased investigation and evidence of strain rate dependent neuronal injury [21, 22], investigation of axonal strain rates might be more useful in this scenario. In this regard, some studies pointed out that axons can accommodate large deformations (up to 100%) under quasi-static loading conditions while rapid deformations might result in brittle nature of the brain tissue—leading to a cascade of microstructural damage scenarios [10,23]. These strain rates might trigger pathological abnormalities leading to secondary dysfunction and damage.

Therefore, to study the behavior of axonal strain rates under blast loading scenarios, we have developed an embedded element based human head finite element model, and intend to use this model in capturing the axonal strain rate response. We are particularly interested in the axonal strain rates caused due to skull flexures (high-frequency skull vibrations resulting from blast wave-head interactions) resulting from exposure to different blast overpressure waves. There has been an increased interest in the study of skull flexures and their effect on the brain response. Recent studies showed that the skull flexures might result in the intracranial pressure surges [8] and could be a possible mechanism of traumatic brain injury [24]. Here, we hypothesize that these high-frequency, low amplitude vibrations might also result in high axonal strain rates and might trigger pathological abnormalities in white matter leading to secondary dysfunction and damage.

This paper is aimed at providing insights into the biomechanics involved in the primary blast exposure to the head by examining the axonal response due to skull flexures as a potential injury mechanism involved in the blast brain injury. The authors are particularly interested in investigating the vulnerability of the human head to skull flexure induced axonal deformations with different overpressures and loading directions. In this direction, a parametric study will be developed. To the authors’ knowledge, this is the first work investigating the axonal response due to skull flexures over a wide range of blast loading conditions using a finite element model of the human head with explicitly embedded axonal tractography. The novelty lies in the use of finite element model with embedded axonal tractography in the investigation of injury biomechanics of human head under blast loading.

## Methods

### Model development

**Fig 1:**
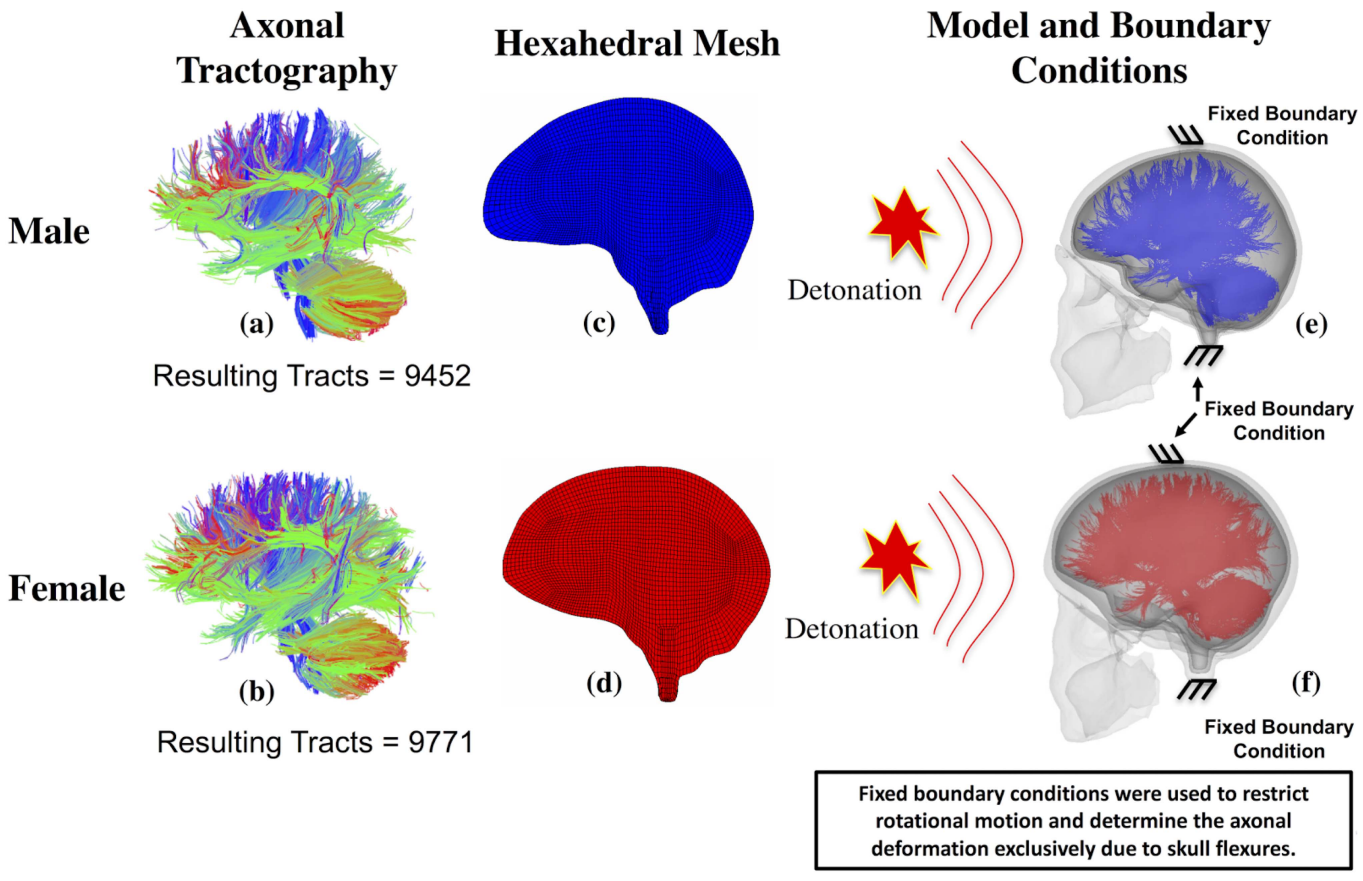
Axonal tractography, corresponding hexahedral brain mesh and the boundary conditions used to investigate the skull flexural effect on axonal deformation. (a) Axonal tractography of the male model with 9452 axonal tracts, (b) Axonal tractography of the female model with 9771 axonal tracts, (c) High-quality hexahedral mesh of the male brain, (d) High-quality hexahedral mesh of the female brain, (e) Male head model with embedded axonal tracts, and (f) Female model with embedded axonal tracts.

Two different embedded element based finite element models of the human head, of male and female individuals (to signify the increasing female population in the military), were developed using the corresponding patient-specific T1 magnetic resonance imaging (T1-MRI) data and diffusion tensor imaging (DTI) data. This medical imaging data was collected after the subjects signed an informed consent form and this protocol is approved by the Institutional Review Board of the Pennsylvania State University (IRB # 30288). The MRI data was used in developing three dimensional anatomical surfaces describing the anatomy of the human head. These surfaces were used in creating high-quality hexahedral meshes–using the multi-block meshing capability in ANSYS ICEM CFD. Fig 1C and 1D shows the high-quality hexahedral meshes belonging to the male and female T1-MRI data, respectively.

Axonal tractography, developed using Diffusion Toolkit (www.trackvis.org/dtk) and TrackVis (www.trackvis.org), was converted into a finite element truss mesh. The resultant tractography, of male and female, has 9452 and 9771 axonal tracts respectively. Each of the truss elements in the axonal fiber mesh was assumed to have a circular cross-section with their diameter representing the diameter of axonal bundles. In this study, we used 1.12 mm as the diameter based on the study by Guy et al. [25]. Fig 1A and 1B shows the axonal fiber tractography developed from patient-specific DTI while Fig 1E and 1F shows the patient specific head models with embedded axonal tractography.

Finite element meshes of the axonal tractography and the head mesh were coupled using the embedded element technique. This is a mesh superposition technique. The hexahedral mesh (of brain tissue) acts as the host mesh and the truss element mesh (of the fiber tracts) acts as the embedded mesh. Once the technique is activated, the nodes of the embedded mesh follow the nodal displacements of the host mesh–resulting in an affine transformation of strains from host to embedded mesh. Eq (1) describes the displacement transfer from host nodes to embedded nodes.

$${\text{u}}_{embed}=[\text{N}]{\text{u}}_{host}$$ (1)

Simultaneously, the stiffness of the embedded mesh (calculated from the material properties of the embedded mesh and the displacement vector) reinforces the stiffness of the host mesh (calculated using the material properties of the host mesh and the displacement vector) resulting in a directional stiffening of the brain tissue (anisotropy). Eq (2) shows the effective internal nodal force vector (analogous to stiffness matrix in small strain domain) i.e., the total stiffness in the model is a sum of independent contributions from matrix and fiber. In this equation,**B** is the strain displacement matrix, σ is the Cauchy stress-tensor, Ω_m_ is the matrix (brain tissue) domain and Ω_f_ is the fiber domain.

$$f_{e}^{int}=\int_{matrix}B^{T}\sigma d\Omega_{m}+\int_{fiber}B^{T}\sigma d\Omega_{f}$$ (2)

The methodology used in the developing the finite element model here is same as that of our recent work [26] (more details can be found here).

### Material properties

Material properties, employed in the models, are listed in Table 1. An isotropic elastic material model was used to describe the skull and skin tissues. Cerebrospinal fluid (CSF) was modeled using a Mie-Gruneisen equation-of-state, with the deviatoric behavior modeled by defining a shear viscosity [14,27–29]. A Mooney-Rivlin hyper-viscoelastic material model was utilized to represent the brain tissue matrix [30,31] and an Ogden hyperelastic material model was used to represent the axonal fibers [30].

**Table 1:** Different anatomic components, material models and corresponding material parameters used in the finite element model. In this table, ρ is the material density,E is the young's modulus, ν is the poisson ratio, C_0_ is the speed of sound, S is the linear Hugoniot slope coefficient, Γ_0_ is the Gruneisen gamma at the reference state, η is the shear viscosity, α is the Ogden material constant, C_01_ and C_10_ are the Mooney-Rivlin material constants, K is the bulk modulus, μ is the shear modulus.

Anatomical Components	Material Model	Material Parameters	References
Skull	Isotropic Elastic	ρ = 1030 kg/m^3^ E = 100 kPa ν = 0.45	[32]
Cerebrospinal Fluid	Mie-Gruneisen Equation of State	ρ = 1000 kg/m^3^ C_0_ = 1489 m/s s = 1.79 Γ_0_ = 1.65 η = 0.001	[33]
Brain Tissue (Matrix)	Mooney-Rivlin Hyperelastic	ρ = 1040 kg/m^3^ C_01_ = -1.034 kPa C_10_ = 7.803 kPa K = 2.1 GPa	[30]
	Viscoelastic	g_1_ = 0.65425 τ_1_ = 0.0066940 g_2_ = 0.0149τ_2_ = 0.15642	[30]
Axonal Fibers	Ogden Hyperelastic	ρ = 1040 kg/m^3^ μ = 35.64 kPa α = 6.101 D = 9.le^-10^ Pa^-1^	[30]

Note that both brain tissue and the axonal fibers are described using homogeneous material properties. The embedded element method, described above, is an element level modification approach instead of a material level modification approach i.e., this embedded element approach calculates anisotropic nodal forces and no explicit custom constitutive material models (linking fibers and brain tissue) were needed to describe the anisotropy.

### Model validation

An extensive validation procedure was adopted here to improve our confidence in the model. Firstly, the models’ response was compared with Nahum et al.’s [34] intracranial pressures from their cadaver experiments. This is followed by a comparison with Hardy et al.’s [35,36] brain-skull relative displacements from their experiments. Finally, the models were subjected to blast loading, and the models' pressure response was compared with Bir et al.’s [37] post-mortem human subject (PMHS) test results. These validation procedures and the corresponding results were included in S1 Appendix. Besides the validation plots, efforts were also made to produce a quantitative assessment of the different validation plots. More details in this regard were include in the following sections.

Once the validation procedure was completed, we went a step further to develop quantitative measures for the above comparison studies. For this purpose, we have used a tool called CORA (acronym for CORrelation and Analysis). CORA can provide us with an objective (and unbiased) assessment of the above validation results [38].

**What is CORA?** CORA is a software tool that calculates the level of correlation between two signals (i.e., time histories). A result lying between “0” and “1” indicates the quality of the match between the signals. “1” represents a perfect match while “0” represents a poor match. This correlation is calculated using metrics such as corridor metric (evaluates deviation between curves) and cross-correlation metric (evaluates size, progression and phase shift of one signal with respect to other).

- Corridor method: This method evaluates the deviation between two signals using a corridor fitting. Four curves (2 curves form a corridor, i.e., two corridors are formed–inner and outer–around the reference curve were formed based on the user defined parameters). A rating (“1” for curve inside inner corridor; “0” for curve outside outer corridor; interpolated rating for curve lying in-between) will be provided to the comparison curve based on its location relative to the corridor positions.
- Cross-correlation Method: It compares the size, phase shift and progression of the comparison curve with respect to the reference curve. The final rating is a weighted normalized rating combining the above three characteristics.

More details can be found here (http://www.pdb-org.com/en/information/18-cora-download.html). Table 2 shows the bio-fidelity score against the CORA ratings.

**Table 2:** Bio-fidelity scale against CORA ratings. A CORA rating should be greater than 0.26 for a comparison to be acceptable.

CORA Rating	Bio-fidelity Scale
0.86–1.00	Excellent
0.65–0.86	Good
0.44–0.65	Fair
0.26–0.44	Marginal
0.00–0.26	Unacceptable

### Loading and boundary conditions

Blast waves are shock waves generated from the detonation of explosives causing rapid heating and expansion of the detonated products. This causes an abrupt compression of the surrounding medium [39].This almost instantaneous detonation generates a propagating shock wave with a discontinuous increase in pressure, temperature, and density [40]. This wave can be approximated using the following Friedlander equation i.e., Eq (3) [41,42].

$$P(t)=P_{s}e^{-a}(1-a)$$ (3)

Here, a = t/t_s_, P_s_ is the peak overpressure and t^*^ is the time at which blast overpressure curve first crosses the time axis. The ConWep (Conventional Weapons Effects program) model implemented in ABAQUS/6.14–2 (Dassault Systems Simulia Corp., Providence, Rhode Island, USA) is used here to simulate the blast loading on the three-dimensional head models. This automates the task of determining the pressure loads applied to surfaces (including oblique surfaces to the incident wave).

**Fig 2:**
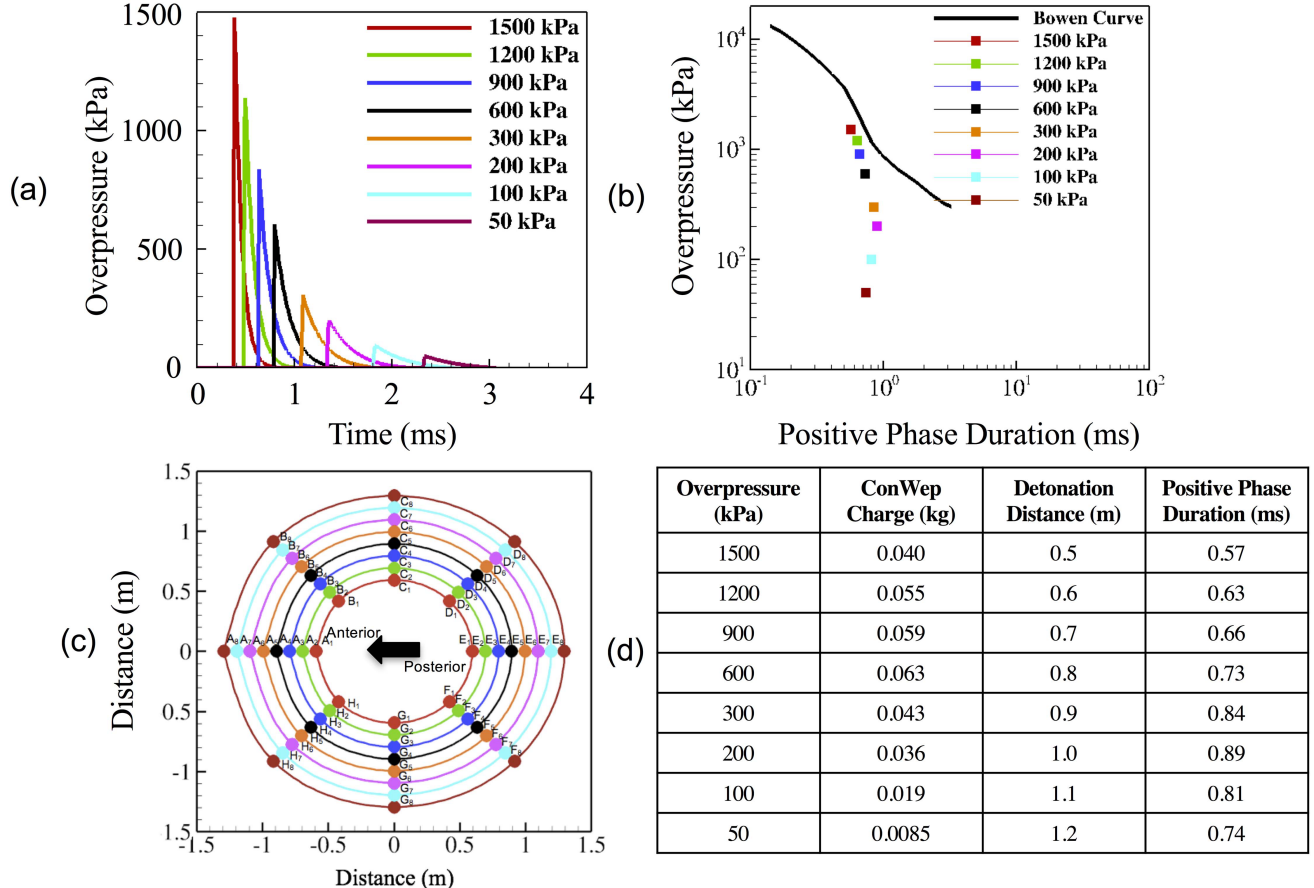
Different loading conditions were used to determine the effect of variation in loading (direction and magnitude) on the axonal response. (a) ConWep blast loading curves. The different loading magnitudes simulated here include 1500 kPa, 1200 kPa, 900 kPa, 600 kPa, 300 kPa, 200 kPa, 100 kPa, 50 kPa. These blast loads are simulated using the ConWep tool in ABAQUS. (b) Blast loading conditions in comparison to Bowen’s lung threshold curve. This plot shows that all the loading conditions opted here fall below the threshold—indicating that the injury will not result in the death of the subject. (c) Arrangement of detonation points around the head form. This arrangement allows us to study the effect of variation in loading direction on the resulting axonal response. (d) Table shows the different ConWep parameters (Overpressure, ConWep charge, Detonation Distance and Positive Phase Duration) for the corresponding loading values used in this paper.

The validated models were subjected to loading conditions accounting for different overpressures. Researchers have used peak loading overpressures ranging from 50 kPa to 1500 kPa while developing the finite element human head blast models [7–9,42–45].In this study, we have used the loading overpressures spanning the entire range of overpressures used by previous researchers. The different peak overpressure values used in this study include 50 kPa, 100 kPa, 200 kPa, 300 kPa, 600 kPa, 900 kPa, 1200 kPa, 1500 kPa. The different overpressure curves were shown in the Fig 2A. Directional variation in the applied loading was achieved through the distribution of detonation points around the head form as shown in the Fig 2C. The simulations for all these different loading conditions were developed and studied to determine the variance in the axonal response and severity under these different input conditions. It was made sure that all the loading conditions used in these simulations fall below the corrected Bowen's lung injury threshold curve [46], as shown in Fig 2B (plotted using Fig 2D). Fixed boundary conditions (zero normal/tangential displacements) were used on the head model to eliminate any rotational effects and the resulting axonal strains as shown in Fig 1E and 1F. Fig 1A and 1B show the male and female head models with embedded axonal tractography. A sample ABAQUS input file (containing the head model subjected to a blast loading) can be accessed here: https://doi.org/10.6084/m9.figshare.5808792

### CSF material model variance

Material properties, used to represent the different head anatomy, are still being studied, i.e., there is no consensus on the accurate material description of the various anatomical components of the head model [47]. The variance in the material description could very well affect the model’s mechanical response to external loading [47]. In this paper, since we were interested in the axonal deformation resulting from flexural displacements of the skull and since CSF forms the interface between brain and the skull, it is a prudent choice to study the effect of variance in the material description of CSF on the axonal deformation. Researchers have used different material models to describe the CSF layer in the head model over the years [33,48–63]. These different material models include (1) linear elastic, (2) visco-elastic, and (3) fluid type modeling (for example Zhou et al. [48] modeled CSF as linear elastic with `fluid' option switched on). Here, the authors used a neo-hookean hyperelastic modeling to simulate the fluid material models used by other studies. Also, a hyper-viscoelastic material model was used to model the viscoelastic material models. The different CSF material models, as well as the corresponding ABAQUS models, are presented in Table 3. Most of the studies presented in the table were collected from a review paper by Dixit et al. [47]. Simulations in this investigation were developed for a frontal overpressure of 1500 kPa. We have chosen this overpressure to extract the peak axonal strains/strain rates for an extreme loading condition used in the parametric study of this manuscript.

**Table 3:** Different CSF material models used in the past literature. These different models used include (i) linear elastic, (ii) non-linear hyper-elastic and hyper-viscoelastic, (iii) fluid. Since CSF forms the interface between skull and the brain, the variance in the CSF material description could affect the model predictions.

Literature	Material Model	Material Parameters	Material Models in ABAQUS	
			Material Model	Material Parameters
Zhou et al. [48,49], Chen et al. [50], Yan et al. [51]	Fluid	ρ = 1040 kg/m^3^ K = 22 MPa G = 50 kPa	Neohookean hyperelastic	ρ = 1040 kg/m^3^ C_1_ = 25 kPa D = 9.1e^-8^ Pa^-1^
Ruan et al. [64]	Fluid	ρ = 1040 kg/m^3^ K = 1.65 MPa G = 50 kPa	Neohookean hyperelastic	ρ = 1040 kg/m^3^ C_1_ = 25 kPa D = 1.2e^-6^ Pa^-1^
Choi et al. [53]	Fluid	ρ = 1040 kg/m^3^ K = 100 MPa ν = 0.5	Neohookean hyperelastic	ρ = 1040 kg/m^3^ C_1_ = 5 kPa D = 2.0e^-8^ Pa^-1^
Willinger et al. [65], Kleiven et al. [55], Ho et al. [56]	Fluid	ρ = 1040 kg/m^3^ K = 2.1 GPa ν = 0.5	Neohookean hyperelastic	ρ = 1040 kg/m^3^ C_1_ = 100 kPa D = 9.5e^-10^ Pa^-1^
Willinger et al. [57,58]	Elastic	ρ = 1040 kg/m^3^ E = 12 kPa ν = 0.5	Elastic	ρ = 1040 kg/m^3^ E = 12 kPa ν = 0.5
Horgan et al. [59,60]	Elastic	ρ = 1040 kg/m^3^ E = 0.15 MPa ν = 0.5	Elastic	ρ = 1040 kg/m^3^ E = 0.15 MPa ν = 0.5
Zong et al. [61]	Elastic	ρ = 1040 kg/m^3^ E = 2.19 MPa ν = 0.489	Elastic	ρ = 1040 kg/m^3^ E = 2.19 MPa ν = 0.489
Mao et al. [62]	Viscoelastic	ρ = 1040 kg/m^3^ K = 2.19 GPa G_0_ = 0.5 GPa G∞ = 0.1 kPa β = 80 s^-1^	Neohookean hyperviscoelastic	ρ = 1040 kg/m^3^ C_1_ = 0.25 kPa D = 9.13e-^10^ Pa^-1^ g_i_ = 0.8 k_i_ = 0 τ_i_ = 0.0125
Yang et al. [63]	Viscoelastic	ρ = 1040 kg/m^3^ K = 1050 MPa G_0_ = 1.0 kPa G∞ = 0.9 kPa β = 80 s^-1^	Neohookean hyperviscoelastic	ρ = 1040 kg/m^3^ C_1_ = 0.5 kPa D = 1.9e^-9^ Pa^-1^ g_i_ = 0.1 k_i_ = 0 τ_i_ = 0.0125
Kleiven et al. [66]	Mie-Gruneisen Equation of State	ρ = 1040 kg/m^3^ C_0_ = 1485 m/s s = 1.79 Γ_0_ = 1.65 kPa η = 0.001	Neohookean hyperviscoelastic	ρ = 1040 kg/m^3^ C_0_ = 1485 m/s s = 1.79 Γ_0_ = 1.65 kPa η = 0.001

A frontal blast loading simulation was developed with the different material models shown in the above table—to study the effects of CSF material model variance on the axonal deformation. A total of 10 simulations were developed in this regard (each referring to a CSF material model). The corresponding axonal deformation was quantified using the maximum strains and strain rates and were presented in the “Results” section.

## Results

### CORA analysis–validation

In this section, we have listed the CORA values obtained for both male and female models under different validation scenarios.

Table 4 shows the CORA ratings calculated for the Intracranial pressure verification against the experiments developed by Nahum et al. [34]. From this table, we can see that all the ratings are above 0.26 making the validation scenarios acceptable.

**Table 4:** CORA ratings for the different intracranial pressure validation plots. Models were subjected to impact loading conditions same as that of the experimental study by Nahum et al. [34].

Comparison Case	Male Model	Female Model
**Frontal Lobe**	0.665	0.660
**Parietal Lobe**	0.711	0.619
**Occipital Lobe**	0.658	0.629
**Overall**	**0.678**	**0.636**

Tables 5 and 6 show the CORA ratings calculated for the brain-skull relative displacement verification against the experiments developed by Hardy et al. [35,36]. Table 5 shows the CORA ratings calculated for the validation plots during frontal and occipital loading conditions while Table 6 shows the CORA ratings calculated for the validation plots during occipital loading conditions. All the CORA ratings in this table, for both the models, can be classified as “good” and “fair” based on the above CORA bio fidelity scale (Table 2). The overall ratings are well above 0.26, making them acceptable.

**Table 5:** CORA ratings for the different brain-skull relative displacement validation plots (impact loading–occipital and frontal). Models were subjected to impact loading conditions, same as that of the experimental study by Hardy et al. [35,36].

Comparison Case	Occipital Impact		Frontal Impact	
	Male	Female	Male	Female
**A1-X**	0.578	0.930	0.434	0.486
**A1-Z**	0.519	0.318	0.332	0.470
**A5-X**	0.346	0.428	0.405	0.471
**A5-Z**	0.514	0.439	0.387	0.478
**P1-X**	0.463	0.919	0.515	0.655
**P1-Z**	0.589	0.431	0.411	0.533
**P5-X**	0.401	0.406	0.539	0.790
**P5-Z**	0.622	0.520	0.619	0.522
**Overall**	**0.504**	**0.549**	**0.455**	**0.551**

**Table 6:** CORA ratings for the different brain-skull relative displacement validation plots (impact loading–parietal). Models were subjected to impact loading conditions, same as that of the experimental study by Hardy et al. [35,36].

Comparison	Male Model	Female Model
**A4-Y**	0.507	0.600
**A4-Z**	0.485	0.514
**A11-Y**	0.387	0.350
**A11-Z**	0.520	0.512
**Overall**	**0.475**	**0.494**

Table 7 shows the CORA ratings calculated for the validation plots during blast loading conditions used by Bir et al. [37]. Some of the CORA ratings in this table, for both the models, can be classified as “good” and “fair” based on the above CORA bio-fidelity scale (Table 2). However, some of the ratings are well below 0.26. This might be due to the huge difference in the results of the two different experiments.

**Table 7:** CORA ratings for the different intracranial pressure validation plots (blast loading). Models were subjected to blast loading conditions, same as that of the experimental study by Bir et al.[37].

Comparison Case	Male Model	Female Model
**71 kPa**		
**Frontal**	0.410	0.594
**Occipital**	0.289	0.365
**Parietal**	0.384	0.473
**76 kPa**		
**Frontal**	0.370	0.400
**Occipital**	0.125	0.130
**Parietal**	0.068	0.080
**104 kPa**		
**Frontal**	0.223	0.227
**Occipital**	0.128	0.140
**Parietal**	0.184	0.190

After the validation, peak maximum axonal strain and peak maximum axonal strain rates were chosen to study the brain response to skull flexures for a given blast loading. These factors have been observed to vary based on the blast overpressure and the direction of exposure. These peak values can be used to estimate the severity of the blast loading condition. A total of 128 simulations were developed for both the male and female models with blast overpressure varying in magnitude and direction. Each simulation took about 5 hrs. on 16 processors to reach 4 ms with an output interval of 0.004 ms–leading to a total of 10,240 simulation core hours.

### Flexural displacements

Before analyzing the axonal deformation for the different loading scenarios, we have tabulated the maximum flexural displacements observed for a loading overpressure of 1500 kPa in different directions. Table 8 shows the magnitude of maximum flexural displacements.

**Table 8:** Table shows the skull flexural displacements for a loading overpressure of 1500 kPa in different directions.

Model/Loading Direction	0^o^ (Frontal)	45^o^ (Left Frontal)	90^o^ (Left)	135^o^ (Left Occipital)	180^o^ (Occipital)	225^o^ (Right Occipital)	270^o^ (Right)	315^o^ (Right Frontal)
**Male**	0.26 mm	0.55 mm	0.70 mm	0.45 mm	0.325 mm	0.40 mm	0.70 mm	0.68 mm
**Female**	0.26 mm	0.36 mm	0.30 mm	0.28 mm	0.24 mm	0.25 mm	0.32 mm	0.41 mm

Here, we observed a maximum flexural displacement of 0.70 mm for the male model for an angle of 90 degrees (loading on left side) and an angle of 270 degrees (loading on right side). For the female model, we have observed a maximum flexural displacement of 0.40 mm at an angle of 315 degrees (loading on right/frontal side).

### Female head model–axonal deformation

#### Axonal strains

**Fig 3:**
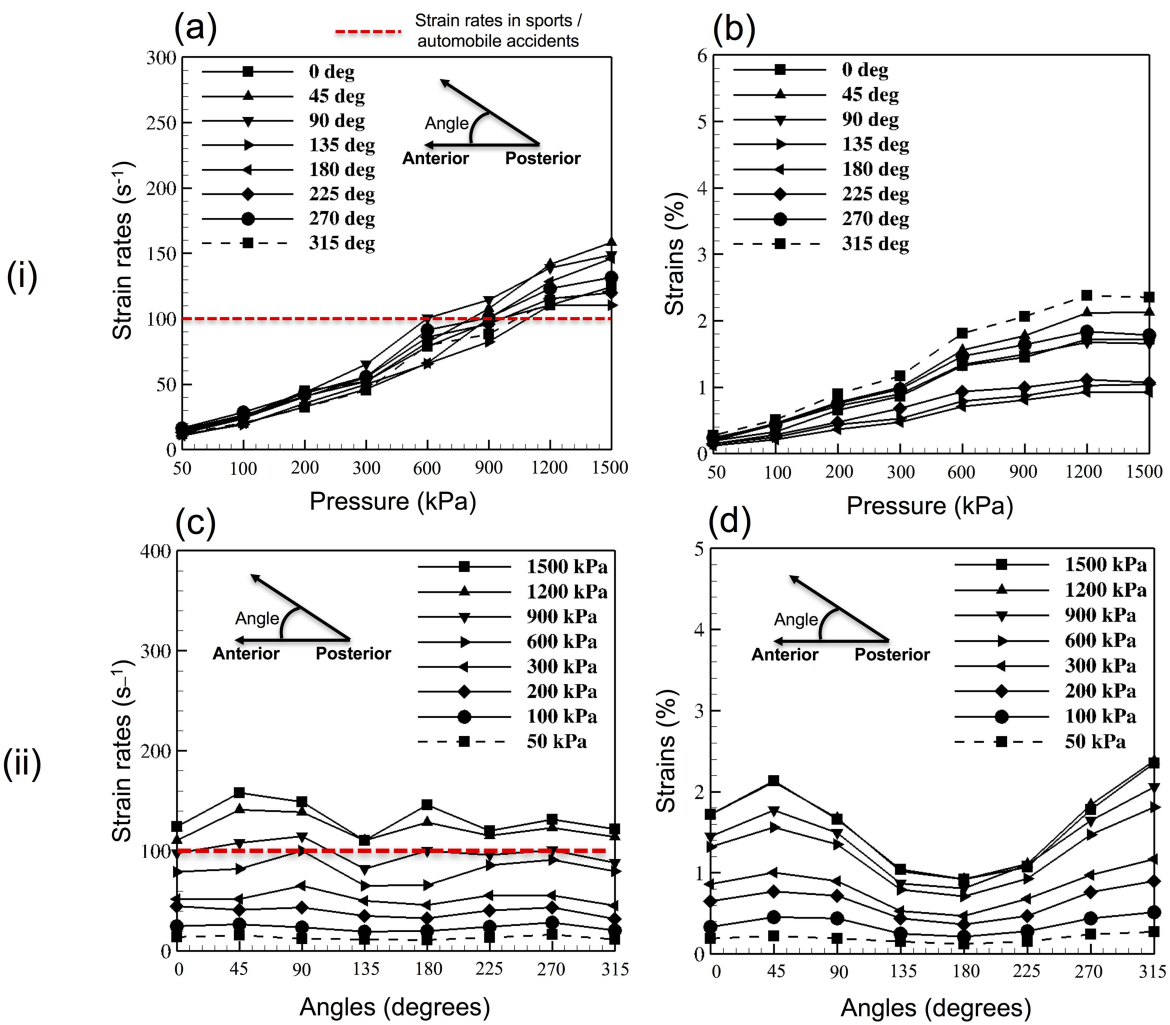
Results from the parametric study conducted on the female head model. (i) Axonal deformation in 64 studies, plotted against different blast overpressure magnitudes–(a) Maximum axonal strain rates, (b) Maximum axonal strains. (ii) Axonal deformation in 64 studies plotted against different blast loading directions–(c) Maximum axonal strain rates, (d) Maximum axonal strains.

Fig 3(i)B and Fig 3(ii)D show the variation in the magnitude of peak axonal strains with increasing blast overpressure and changing blast loading direction. A maximum axonal strain of 2.4% (approx.) was observed for a blast overpressure of 1200 kPa with the loading in left/frontal to right/occipital (angle of 315 degrees) direction. The minimum axonal strain of 0.1% (approx.) was observed for a blast overpressure of 50 kPa.

#### Axonal strain rates

Fig 3(i)A and Fig 3(ii)C show the variation in the magnitude of peak axonal strain rates with increasing blast overpressure and changing blast orientations. A maximum axonal strain rate of 150 s^-1^ (approx.) was observed for a blast overpressure of 1500 kPa with the loading in right/frontal to left/occipital direction. The minimum axonal strain rate of 10 s^-1^ (approx.) was observed for a blast overpressure of 50 kPa.

### Male head model–axonal deformation

#### Axonal strains

**Fig 4:**
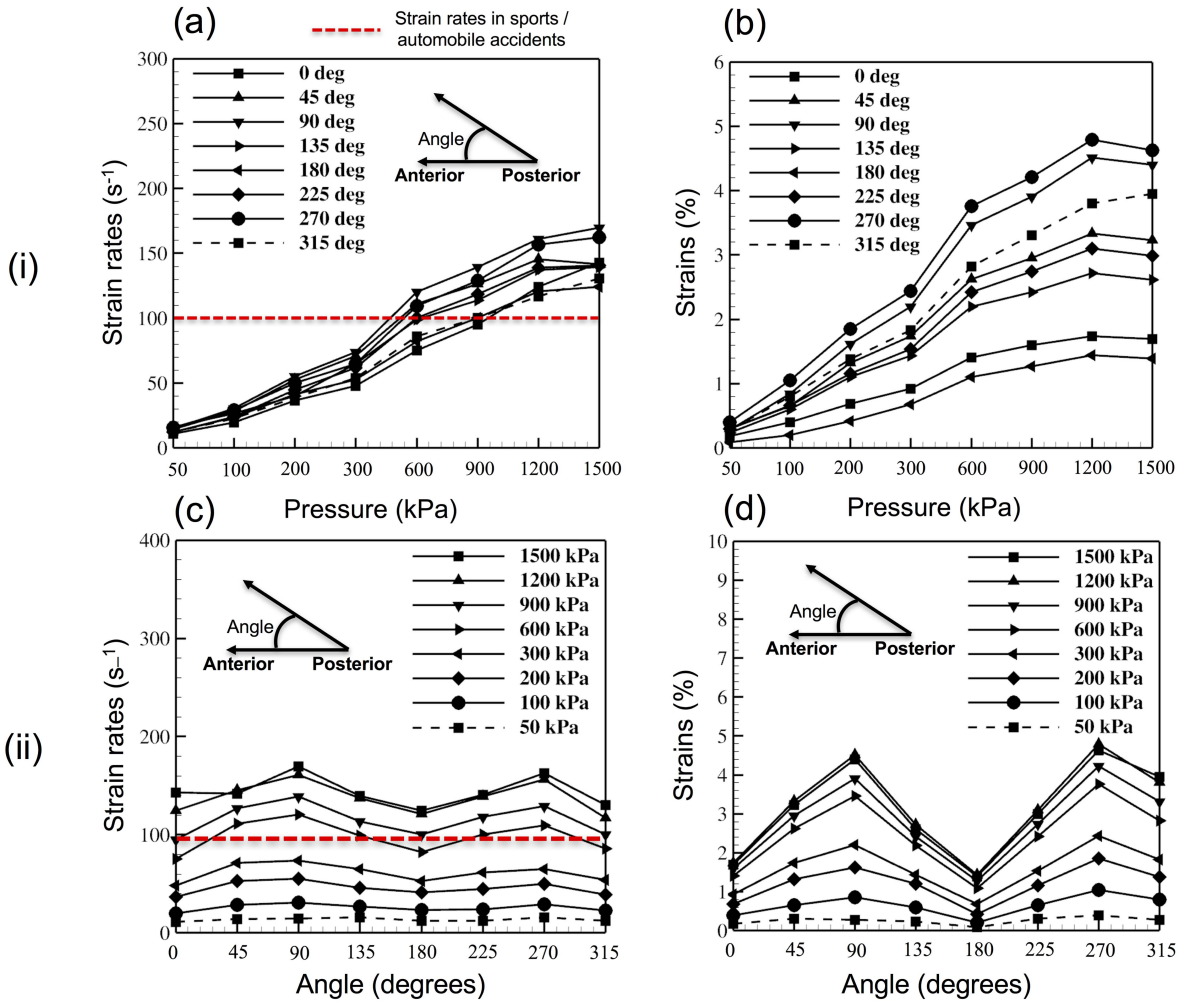
Results from the parametric study conducted on the male head model. (i) Axonal deformation in 64 studies, plotted against different blast overpressure magnitudes–(a) Maximum axonal strain rates, (b) Maximum axonal strains. (ii) Axonal deformation in 64 studies plotted against different blast loading directions–(c) Maximum axonal strain rates, (d) Maximum axonal strains.

Fig 4(i)B and Fig 4(ii)D show the variation in the magnitude of peak axonal strains with increasing blast overpressure and changing blast loading direction. A maximum axonal strain of 5% (approx.) was observed for a blast overpressure of 1200 kPa with the loading in left-right (angle of 270 degrees) direction. The minimum axonal strain of 0.1% (approx.) was observed for a blast overpressure of 50 kPa.

#### Axonal strain rates

**Fig 5:**
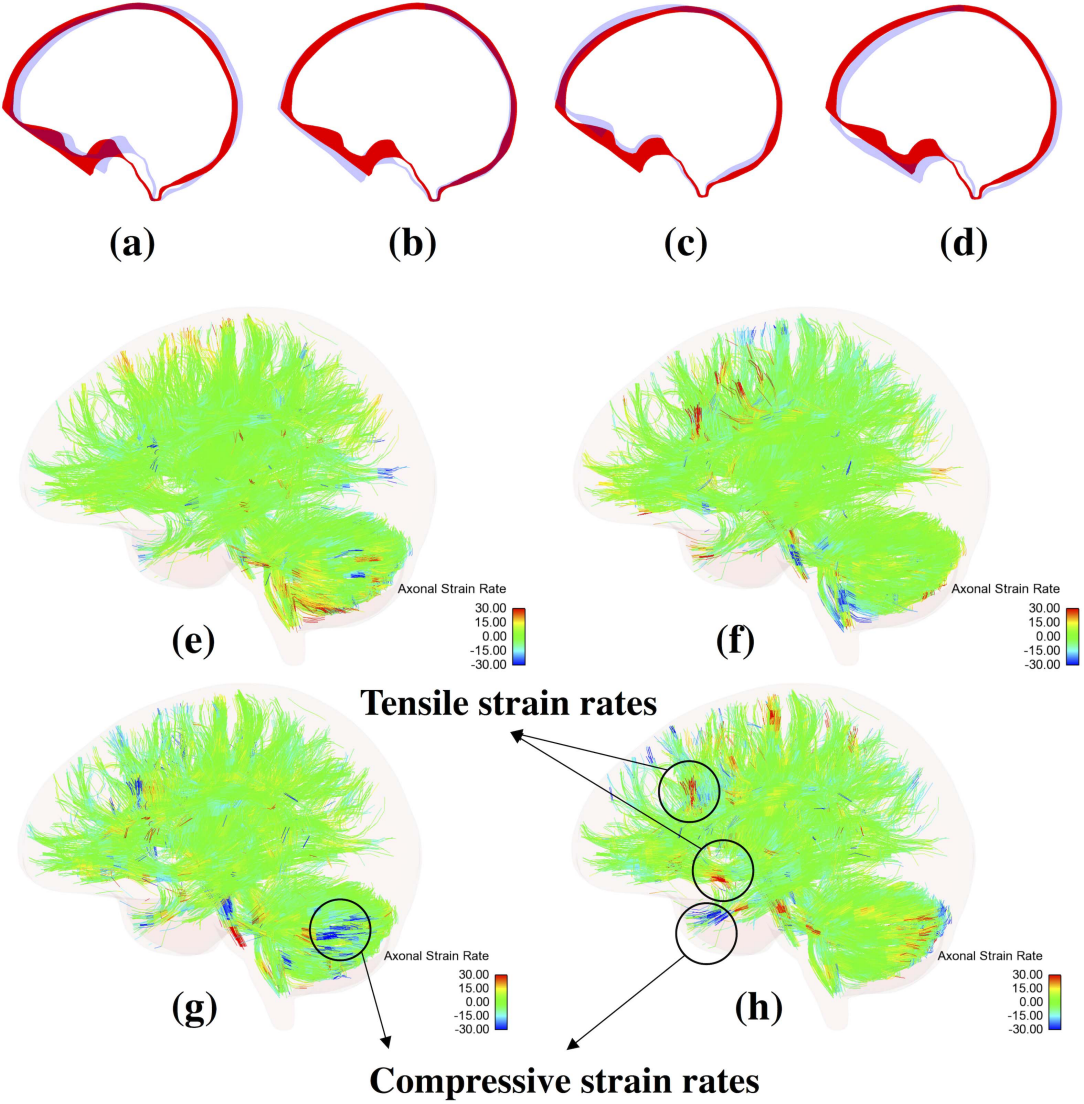
Flexural bending displacements experienced by the skull and the resulting strain rates experienced by the axonal fiber tracts for an overpressure loading of 600 kPa in anterior-posterior direction. A maximum axonal strain rate of 80 s^-1^ was observed in this scenario. This figure also emphasizes the fact that embedded element based head model allows for a high-resolution visualization of the model. (i) Flexural displacements of the skull at (a) t = 1 ms. (b) t = 2 ms. (c) t = 3 ms. (d) t = 4 ms. (ii) Strain rates experienced by the axonal fiber tracts (e) t = 1 ms. (f) t = 2 ms. (g) t = 3ms. (h) t = 4ms. Here, the red color cross-sectional view of the skull represents the original skull shape while the blue color cross-sectional view represents the displaced skull’s cross-sectional view.

Fig 4(i)A and Fig 4(ii)C show the variation in the magnitude of peak axonal strain rates with increasing blast overpressure and changing blast orientations. A maximum axonal strain rate of 170 s^-1^ (approx.) was observed for a blast overpressure of 1500 kPa with the loading in right-left direction. The minimum axonal strain rate of 10% was observed for a blast overpressure of 50 kPa. Fig 5 shows the flexural displacement and the corresponding axonal strain rates experienced by the axonal fibers for a loading overpressure of 600 kPa.

### CSF material variance and resulting axonal deformation

**Fig 6:**
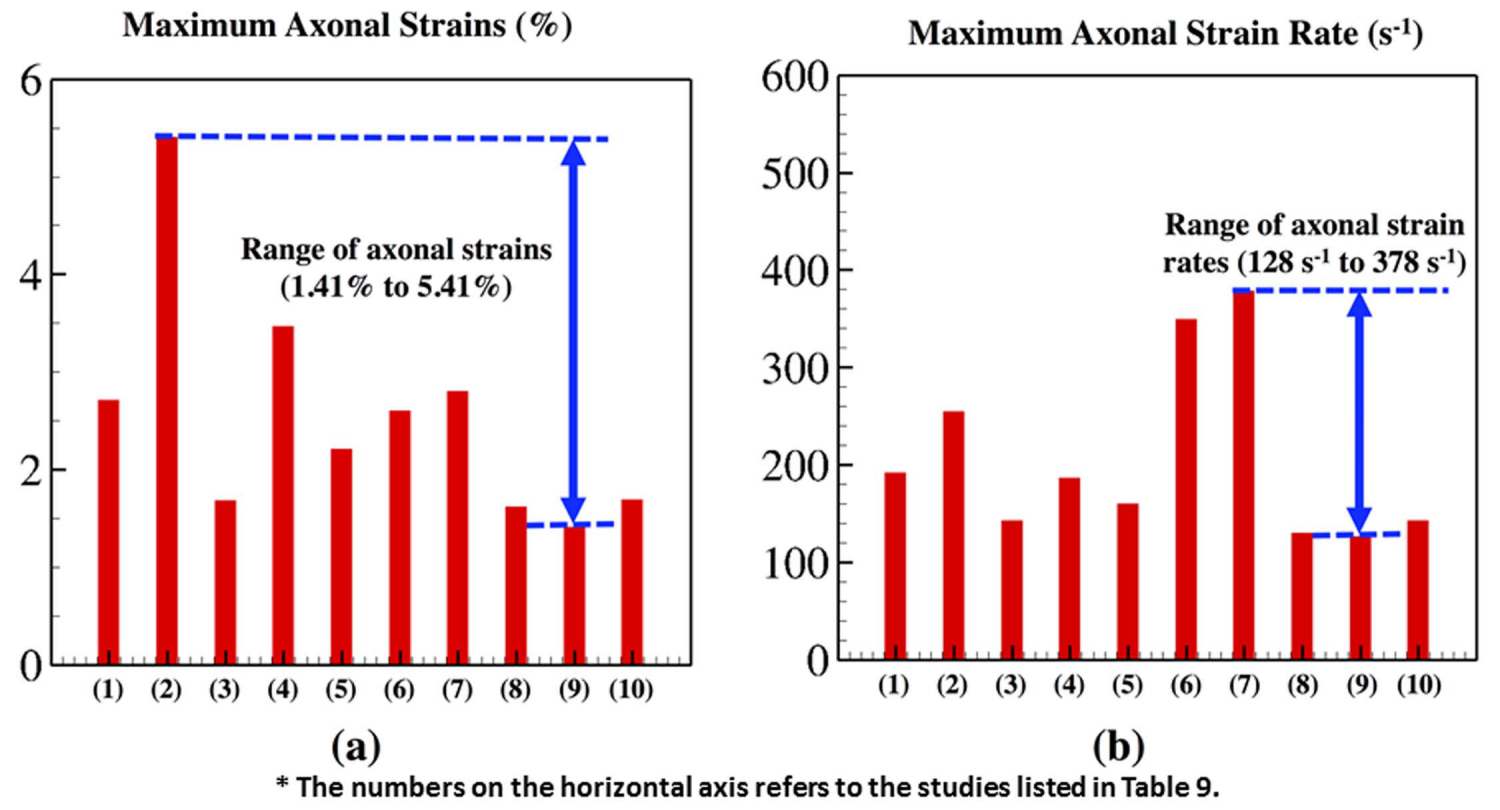
Peak axonal strains and strain rates, for different CSF material models, plotted using a bar graph. (a) Maximum axonal strains (%) observed for the different simulations, (b) Maximum axonal strain rates observed for the different simulations. The horizontal axis in the above plots show the study number assigned in the Table 3. These are the maximum axonal strains and strain rates plotted for different simulation scenarios. These different simulations varied in terms of CSF material description. This study was developed to determine the range of extreme strains and strain rates possible due to skull flexures. The above results show that the maximum axonal strains span a range of 1.41% to 5.41%; and the maximum axonal strain rates span a range of 128 s^-1^ to 378 s^-1^.

The maximum axonal strains and strain rates for the different CSF material descriptions in a frontal blast loading simulation were shown in Table 9. A total of 10 simulations were developed in this regard—a total of 462 core hours. The different CSF material descriptions resulted in different axonal strains/strain rates. Ruan et al.'s [64] fluid type CSF model resulted in the maximum axonal strain of 5.41% while Yang et al.'s [63] viscoelastic material model resulted in the minimum axonal strain of 1.41%. Zong et al.'s [61] linear elastic CSF model resulted in the maximum axonal strain rate of 378 s^-1^ while Yang et al.'s viscoelastic material description resulted in the minimum axonal strain rate of 126.1 s^-1^. Fig 6 shows the axonal strains and strain rates observed for the wide range of CSF material models.

**Table 9:** Table showing the maximum axonal strains and strain rates using different CSF material descriptions. A frontal blast loading simulation of the model head model was developed with the different CSF material descriptions and used it to tabulate the above results.

Study No.	Study	Maximum axonal strain (%)	Maximum axonal strain rate (s^-1^)
1	Zhou et al. [48,49], Chen et al. [50], Yan et al. [51]	2.71	191.7
2	Ruan et al. [64]	5.41	254.6
3	Choi et al. [53]	1.68	142.7
4	Willinger et al. [65], Kleiven et al. [55], Ho et al. [56]	3.47	186.4
5	Willinger et al. [57,58]	2.21	160.4
6	Horgan et al. [59,60]	2.60	349.3
7	Zong et al. [61]	2.80	378.9
8	Mao et al. [62]	1.62	130.2
9	Yang et al. [63]	1.41	126.1
10	Kleiven et al. [66]	1.69	142.9

## Discussion

Two different detailed finite element models, of the male and female human head, were used in this investigation of the axonal fiber tract response to a wide range of blast overpressures and directions. We chose to include both the models because of the increasing female population in the battlefield environment [67]. We believe that the gender differences should also be properly understood, to arrive at true findings of TBI research. Besides this, some of the previous studies have shown that these gender differences could impact the design of protection measures [68]. For example, men and women have different skull thickness with women having a higher average skull thickness compared to that of men [68]. This could result in lower flexural displacement in women under the same loading conditions—leading to lower axonal response. Embedded element method was used to implement the corresponding patient-specific diffusion tractography model into both the finite element models. This technique enables us to implement the fiber information explicitly and in a more accurate way–eliminating the need for an averaged fiber description (when there is more than one fiber per element [69]) or averaged fractional anisotropy per finite element in the white matter. A more detailed discussion on the application, advantages and limitations associated with the use of embedded element method in developing a high-resolution model of the human head was included in our recent work [26]. In this paper, we have focused exclusively on the axonal deformation resulting due to blast-induced skull flexures. A flexure can be defined as the bending or curving or the action of being bent or curved. During the blast event, a unique characteristic event related to the brain injury is the rapid deformation of the skull resulting in tensile and compressive deformation of the brain.

In this paper, we hypothesized that these rapid oscillations of the skull could result in the rapid deformation of the axonal fiber tracts. Upon blast impact, the corresponding oscillation of the skull results in deformation of the brain—thus resulting in the deformation of the axonal fiber tracts. These are high frequency and low amplitude oscillations resulting in large strain rates for small strain magnitudes in the axonal fiber tracts. We have designed a parametric study to investigate this axonal deformation for a wide range of loading conditions (varying in direction and magnitude). This axonal deformation was quantified using the axonal strain/strain rate values. This strain/strain rate is the mechanical response of the axonal fibers i.e., the strain/strain rate measured in trusses along each of the explicitly modeled axonal fiber tract. Furthermore, these strains/strain rates are calculated from the affine transfer of nodal displacements from the host brain tissue and requires no post-processing efforts (as shown in Equation (1)). The authors believe that this axonal mechanical response is more physiologically relevant, and therefore, more accurate for investigating the blast-induced axonal injury. For the simulations, we have used fixed boundary conditions to eliminate the axonal strains/strain rates that might be originating from the rotational deformation of the human head. This investigation might help us study the axonal deformation resulting exclusively from skull flexures. The neck anatomy is also excluded from the model as we are not interested in the axonal deformation resulting from the kinematics of the human head. Axonal strains and strain rates–caused exclusively due to the skull flexures–were documented, in all these simulations.

**Fig 7:**
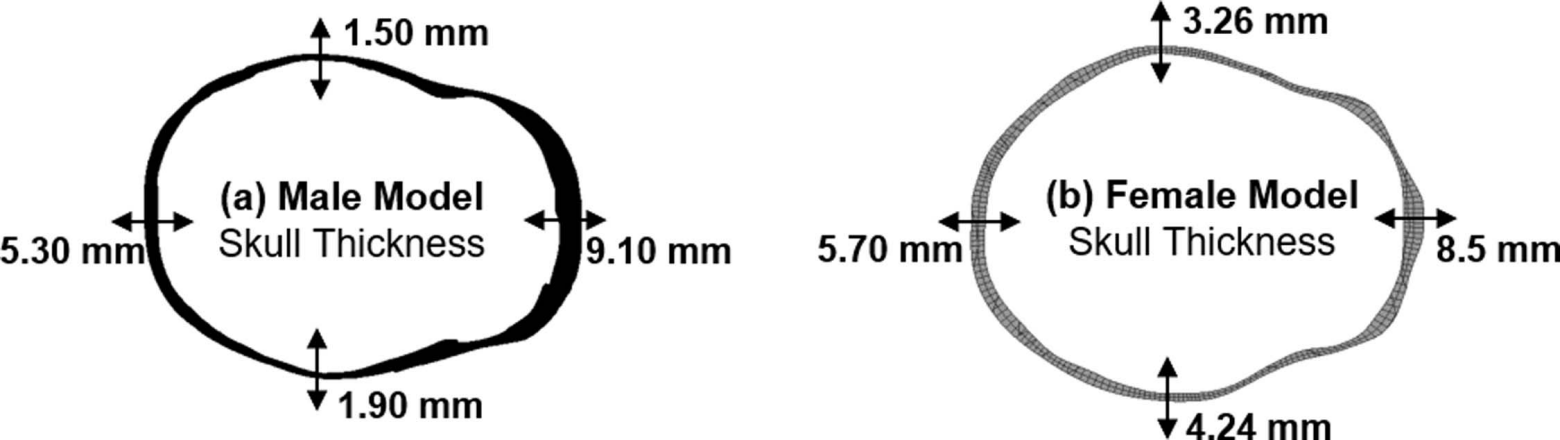
kull thickness across a plane of cross-section for both male and female FE models. **S** The variation in skull thickness could be the reason for different flexural behavior across gender.

For the loading overpressure of 1500 kPa in different directions, flexural displacements seem to be more dependent on the skull thickness. Fig 7. shows the skull thickness in a cross-sectional plane in different regions of the male and female models. For both models, frontal loading resulted in flexural displacement of similar magnitude owing to the similar skull thickness in this region. However, as we move from frontal to the side of the head, maximum flexural displacements of 0.70 mm and 0.40 mm was observed for male and female models, respectively. In the male model, loading in left and right (side impacts) directions seem to result in a higher flexural displacement. This can be explained by the fact that the skull thickness in these regions are low as seen in Fig 7. For the female model, we observed a maximum flexural displacement of 0.40 mm for loading in the left-frontal and right-frontal directions. These observations seem to follow the hypothesis that the skull flexural displacements are dependent on the skull thickness. Besides this, over the different loading conditions, maximum axonal strains and axonal strain rates were primarily dependent on the blast overpressures (and resulting flexural displacements). For example, for both female and male models, strains and strain rates increased with increasing blast overpressure. The axonal response is mainly driven the skull deformation in their respective scenarios. Different peak axonal strains/strain rates were observed for both the male and female models–pointing out to the differences in axonal structure, axonal orientation, and skull thickness. For example, it can be observed that the strain/strain rate magnitudes are higher in simulations of the male head model compared to that of the female head model. This could be a result of the higher skull thickness (average) of the female head model compared to that of the male head model (also evident from Fig 7). This difference also shows the importance of developing patient-specific personalized finite element head models while studying the axonal injury. From the results, it can be observed that the loading in the posterior-anterior direction (blast loading on the back of the head) seems to be the least dangerous scenario as low peak axonal strain and strain rate were observed in this scenario (see Figs 3 and 4).

Furthermore, as the loading overpressure increased, variation in the peak strains/strain rates (for loading in different directions) increased. This behavior is more evident for the male model in the axonal strain behavior. This could be attributed to the huge variation in the skull thickness from frontal (0^o^) to side (90^o^) for the male model i.e., skull thickness reduced from 9.10 mm to 1.95 mm in the male head model when compared to a reduction from 8.5 mm to 3.26 mm in the female head model. This also implies that the axonal strains are more dependent on skull thickness when compared to the axonal strain rates and that the blast loading direction plays an important role for higher overpressures. Another possible reason for this noticeable variation in axonal strains might be the orientation/arrangement of the different axonal fiber tracts. This plays an important role in the evolution of axonal damage this varies from person to person and across gender [70]. For example, in our previous publication [26], we have shown that the axonal damage for a particular loading condition is dependent on the orientation of the axonal fiber tracts. Here, even though the axonal strains observed are much lower in magnitude (<5%), very high strain rates were observed (**≈**170 s^-1^). These strain rates are well above the strain rates usually seen in impact loading situations (sports/ automobile accidents, **≈**100s^-1^ [10]) and the strain rates usually adopted in experimental studies aimed at developing axonal injury thresholds.

Besides this, simulations were also developed using the different CSF material models to determine the maximum axonal strains and strain rates. This study was developed to investigate the effect of CSF material model invariance on the model response. The different CSF material models resulted in different axonal strains ranging from 1.4% to 5.4% and a wide range of axonal strain rates spanning 120 s^-1^ to 378 s^-1^. Based on these results, we can conclude that the CSF material model used in our first parametric study resulted in a conservative estimate of the axonal deformation in different scenarios. That study will be improved in the future by introducing the CSF material model as another variable into the parametric study. Besides this, this study also provides us with a possible range of strains/strain rates resulting from the flexural displacements of the skull and might provide us with some new insights into the predictive capabilities of the head model. The strain rates, as high as 378 s^-1^, strengthens our argument that there is a huge possibility of micro-structural damage due to the flexural displacement of the skull [71]. These results also strengthen the argument that a consensus on the accurate material description of the different human head anatomy needs to be achieved before we can develop an accurate blast based predictive injury models of the human head.

There is no consensus on the best injury criteria for axonal damage–making it difficult for us to arrive at a conclusion on the flexure induced axonal damage using the above results. In this paragraph, we have reviewed the available injury criterion and attempted to connect it back to our results. There are wide variations in the existing proposed injury criterion. Different anatomical models were used to determine these thresholds. Some used non-mammalian optic nerves and squid giant axon models while some used mammalian spinal nerve roots [70,72–75]. Strain thresholds varied from 5% to 65% in the data. These different strain thresholds are determined at different strain rates (18 s^-1^ to 100 s^-1^) [21,70,72–75]. Few of the studies did not include the injury dependence on strain rates. Some studies have shown that the strain thresholds decreased with an increase in the applied strain rate. However, these studies performed experiments up to a very low strain rate values. For example, Morrison et al. [76] performed experiments for a large range of strains only up to 50 s^-1^ strain rate. However, strain rates as large as 378 s^-1^ are observed during the blast loading scenarios here. Therefore, conclusions about injury can only be made when the empirical data for damage of axons at low strains and strain rates as high as 170–200 s^-1^ is available. One interesting direction of investigation of axonal damage is the microstructural damage resulting from rapid axonal deformation. There have been some recent studies stressing the importance of strain rates and their role in the occurrence of neuronal injury. For example, Ahmadzadeh et al. [77] reported the critical strain rates as 22–44 s^-1^ for disassociation of microtubule-associated tau proteins. They explained this based on the strain-stiffening effect experienced by the tau proteins–thus leading to a significant load transfer onto the microtubules and thus microtubule damage. Another study by Bar et al. [22] supported these results by reporting (qualitatively) the neuronal morphological changes (and subsequent bleb formation) at high strain rates. This study also reports a possible rate dependent neuronal injury under compression–which warrants an investigation into the tractography regions experiencing significant compressive strains/strain rates. These above studies suggest that the observed axonal deformation–in our simulations–might result in microstructural axonal damage.

There are certain **limitations** associated with this study. The first limitation is the use of loading conditions. Even though the overpressures are somewhat representative of real world loading conditions, the blast loading duration was less than 2ms. 2–6 ms duration blast loadings represents the real-world conditions [78]. The ConWep charge parameter used here also doesn't represent the real-world situations (<1Kg). The second limitation is the exclusion of internal anatomical components such as falx cerebri, tentorium cerebelli, corpus callosum and ventricles (and corresponding material heterogeneity in the brain). Previous literature reported some differences in the material properties among these different anatomical components. This variation in material properties might create some internal hotspots (less stiff regions might experience more axonal strains) which are not visible right now due to lack of resolution in the model. At the same time, since we are only studying the effects of blast-induced skull flexures—which were more evident near the brain-CSF skull interface, we are cautiously confident that the exclusion wouldn’t make a significant difference in the conclusion provided here. Another limitation is the absence of skin tissue in the finite element model. The presence of skin might attenuate the pressure wave and disperse the loading area thus dispersing the shock load concentration on the skull. This might reduce the magnitude of skull flexures locally resulting in reduced axonal strains and strain rates. Also, validation of the skull deformation (in blast-induced skull flexure scenarios) might be more useful in establishing the validity of the observations. For this purpose, more empirical data on the deformation of skull exposed to blast loading are needed. Besides this, here, skull was modeled as a homogeneous material. This approach was adopted to match the modeling procedure adopted by other previous studies, investigating skull flexures, by Moss et al. [8] (a well cited computational model of skull flexures). At the same time, instead of investigating the intracranial pressure response similar to the work done in the previous studies, we chose to investigate the axonal strains/strain rates. In real life, skull is softer in comparison i.e., it resembles a sandwich structure with a stiff cortical bone on the faces and a softer trabecular bone in the middle. Therefore, the axonal deformation (axonal strains/strain rate magnitudes) presented in this manuscript might be conservative compared to that of the real-life scenario. This will be addressed in our future work. Another area of interest is the arrangement of detonation points around the human head model. Due to the elliptical shape of the human head and the circular arrangement of the detonation points around it, there might be a small error associated with the analysis due to the difference in the distances of the surface from the detonation point in anterior-posterior and left-right directions. However, this difference is small compared to the actual detonation distance, and thus this is neglected.

Aside from the above improvements, some recommendations for the future include an investigation into the frequency response of the skull and axonal fibers under the blast loading scenarios. This study would provide us with insights into the resonant frequencies of the anatomical components–thus enabling us to develop better protection measures under high-frequency loading scenarios such as blast or shock. Also, the authors will enhance the model performance by improving the anatomical resolution of the model. High anatomical resolution can be achieved either by segmentation of the brain mesh into different brain regions or the segmentation of the axonal tractography mesh or by including new anatomical detail such as cerebral vasculature or by doing all the above. This type of modeling effort might enable us to develop a more extensive analysis of the region-wise brain response under these different blast loading scenarios–thus providing us with a region-wise vulnerability map of the brain—which can be a crucial tool in clinical diagnosis. Apart from this, here, the authors have used two different head models (male and female) to investigate the axonal response. Observations suggest that the magnitude of axonal response in the female model is less compared to that of the male model (possibly due to the differences in skull thickness). This effort is a first step towards more comprehensive parametric study with gender difference as another parameter. We will also carry out an in-depth investigation of the differences in axonal deformation in different gender-based models. This might provide us with new insights into the role of gender differences in the evolution of axonal damage–might provide us with new perspectives into a more efficient design of gender-based protective measures. Besides this, the axonal deformation measures (strains and strain rates) developed here will be used as inputs to microscale models to investigate the evolution of potential microstructural damage [2]. Besides using the model to study the blast-induced axonal injury, an effort towards the clinical validation of this fiber based axonal injury model would strengthen our confidence in the prediction capabilities of this model [79]. Also, the explicit modeling (explicitly discretized mesh) of the axonal fiber tracts might enable us to solve a wide variety of partial differential equations–allowing us to solve multi-physics such as diffusion, electro-physics, etc.—thus acting as a linchpin in connecting multiple modeling approaches and enabling us to develop an all-inclusive model [80].

## Conclusion

The focus of this work was to investigate the axonal response due to skull flexures under blast loading. Embedded element method was applied to incorporate the axonal fiber tractography (obtained from DTI) into the FE model of the human head. The model was then used in investigating the skull flexure induced axonal deformation, and determining the directional effects of loading on the axonal response due to skull flexures. Based on the simulation results/observations, it can be concluded that the skull flexure-induced axonal strains are very low in magnitude (< 5%). However, the axonal strain rates go as high as 150–200 s^-1^. These high-axonal strain rates could lead to microscale damage as pointed out by other literature. Also, there is a significant variation (140 s^-1^ to 350 s^-1^) in the axonal strain rates/strains with a variation in the CSF material models–emphasizing the importance of more research into the accurate description of CSF in finite element models.

## Supporting information

S1 Appendix Appendix explaining the supporting information tables and figures. (PDF)

S1 Table Different experimental studies used to verify the accuracy of the head models. (PDF)

S1 Fig Male head model–Intracranial pressure validation against Nahum’s experiments. Comparison of experimental and simulated intracranial pressures for Nahum’s frontal loading condition. Validation plots of the female model were not presented here and included in our previous publication [26].
(PDF)

S2 Fig Male head model–Brain-skull relative displacement validation for frontal impact. Comparison of experimental and simulated brain-skull relative displacements for frontal loading condition (C383T1)–reported by Hardy et al. [35,36].
(PDF)

S3 Fig Male head model–Brain-skull relative displacement validation for occipital impact. Comparison of experimental and simulated brain-skull relative displacements for occipital loading condition (C755T2)–reported by Hardy et al. [35,36].
(PDF)

S4 Fig Male head model–Brain-skull relative displacement validation for parietal impact. Comparison of experimental and simulated brain-skull relative displacements for parietal loading condition (C393T4)–reported by Hardy et al. [35,36].
(PDF)

S5 Fig Female head model–Intracranial pressure response for different overpressure loadings– 71 kPa, 76 kPa and 104 kPa and its comparison with the Bir’s [37] PMHS experimental readings. (PDF)

S6 Fig Male head model–Intracranial pressure response different overpressure loadings– 71 kPa, 76 kPa and 104 kPa and its comparison with the Bir’s [37] PMHS experimental readings. (PDF)
