# Supplementary material for: Do blast induced skull flexures result in axonal deformation?
Source: PLoS One. 2018 Mar 16;13(3):e0190881. doi: 10.1371/journal.pone.0190881 (PMC5856259; doi:10.1371/journal.pone.0190881)
Supplement: S1 Appendix — (PDF) [file pone.0190881.s001.pdf]

S1 Appendix

Once the models were developed, confidence in the finite element models (male and female) and material properties was established by comparison of the results from these models to the experimental studies performed using real cadaver head models. Different studies were used in this regard. These different tests and their classification (in terms of loading and model response) is shown in Table in S1 Table.

S1.1 Validation – impact loading

For pressure verification, one of the six cases from a study by Nahum et al. [34] – a frontal impact case, i.e., Case 37 – was used to verify the predicted brain pressure in the model. The intracranial pressures in the brain tissue region were calculated by averaging the pressures over certain number of elements. Averaging the pressures might give us a more generalized pressure response of the region. Figure in S1 Fig shows the validation plots for this scenario.

For displacement verification, brain-skull relative displacement data from Hardy et al. [35, 36] was used to verify the predicted displacements from the simulation. The displacements were extracted at specific nodes in the model selected based on the location of NDT's reported by Hardy et al. [35, 36]. Figures in S2 Fig, S3 Fig and S4 Fig show a comparison of the brain-skull relative displacement curves for the male head model over time with the experimentally observed results for frontal (C383T1), occipital (C755T2), and parietal (C393T4) impacts published by Hardy et al. [35, 36].

S 1.2 Validation – blast loading

In this section, the female and male models were subjected to experimental blast loading conditions. The models' pressure response was compared to the post mortem human subject (PMHS) tests conducted by Bir [37]. These tests used a shock tube simulated blast wave and reported the corresponding cadaveric human head intracranial pressure response. These tests used overpressures of 71 kPa, 76 kPa and 104 kPa respectively. Here, the model response was quantified by measuring the intracranial pressure response at locations similar to that of the experiments. The corresponding validation results were shown in Figures in S5 Fig and S6 Fig.
