# Supplementary material for: Do blast induced skull flexures result in axonal deformation?
Source: PLoS One. 2018 Mar 16;13(3):e0190881. doi: 10.1371/journal.pone.0190881 (PMC5856259; doi:10.1371/journal.pone.0190881)
Supplement: S1 Table — (PDF) [file pone.0190881.s002.pdf]

**Case**	**Loading Type**	**Model Response**
Hardy et al. [32, 33]	Impact	Brain-skull relative displacement
Nahum et al. [31]		Intracranial pressure
Bir et al. [34]	Blast	
