# Supplementary figures and images for: Do blast induced skull flexures result in axonal deformation?
Source: PLoS One. 2018 Mar 16;13(3):e0190881. doi: 10.1371/journal.pone.0190881 (PMC5856259; doi:10.1371/journal.pone.0190881)

# Frontal Blast Loading – Male Model

**71 kPa**

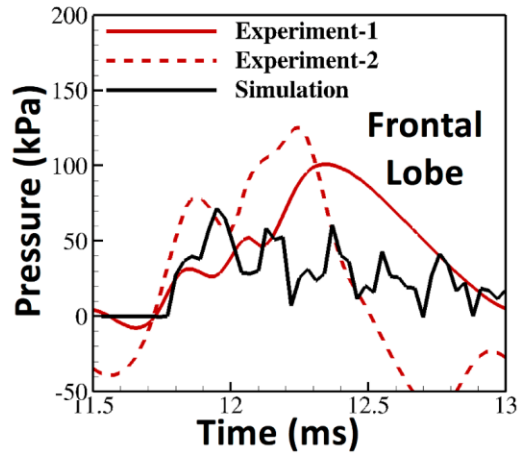

**76 kPa**

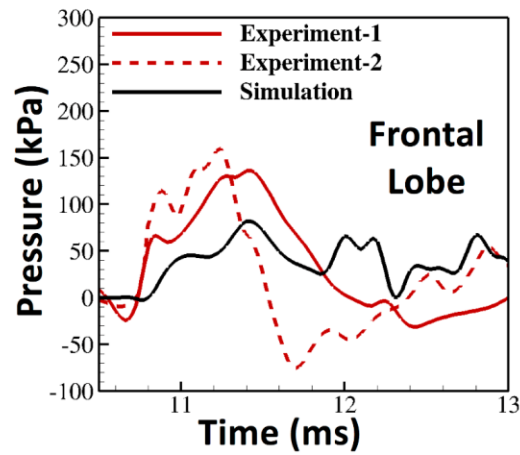

**104 kPa**

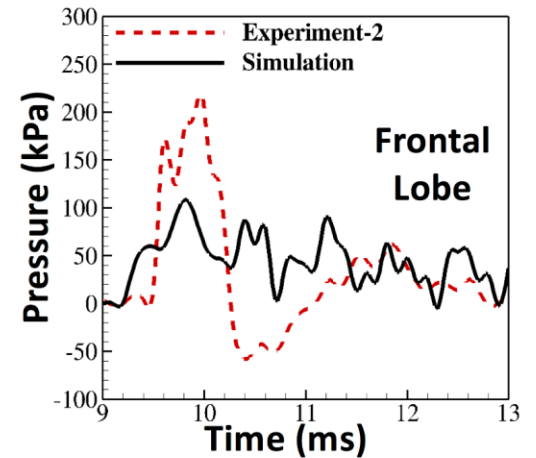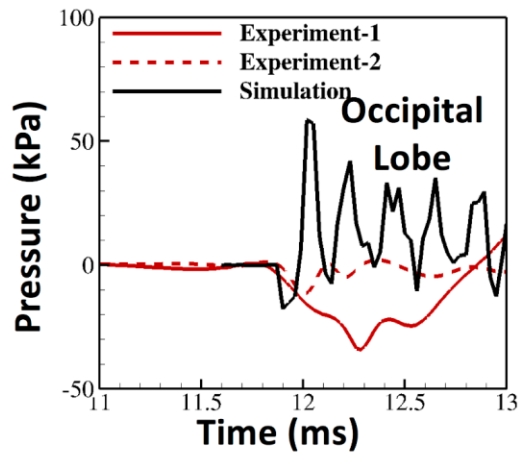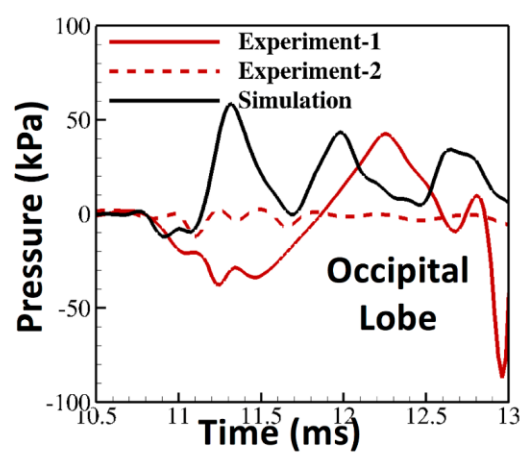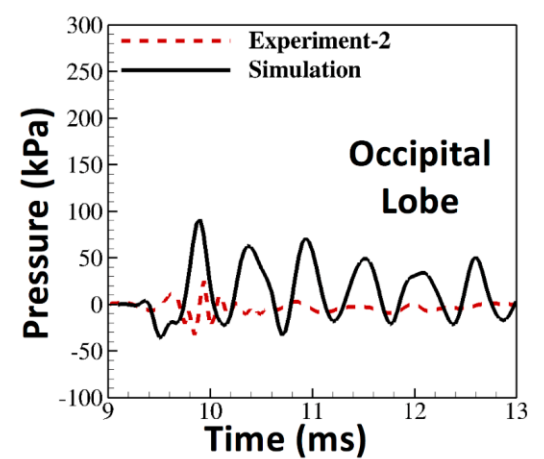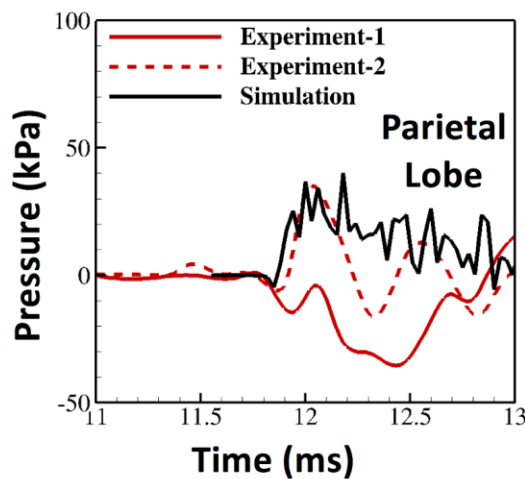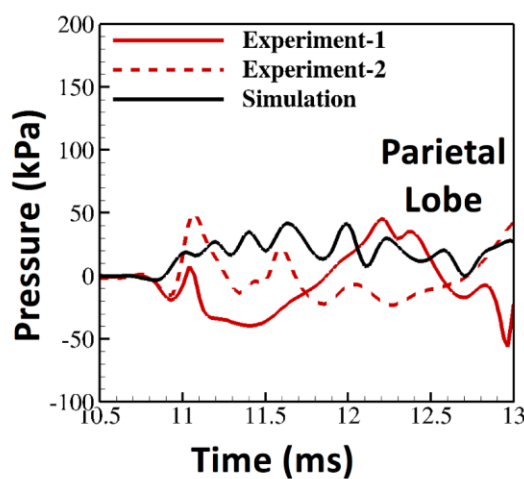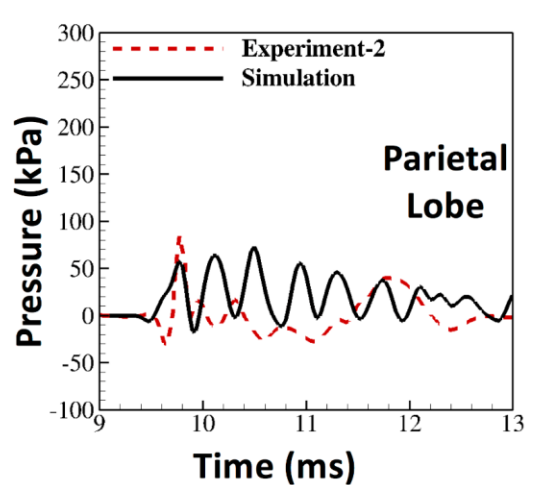

Supplement: S1 Fig — Comparison of experimental and simulated intracranial pressures for Nahum’s frontal loading condition. Validation plots of the female model were not presented here and included in our previous publication [26]. (PDF) [file pone.0190881.s003.pdf]

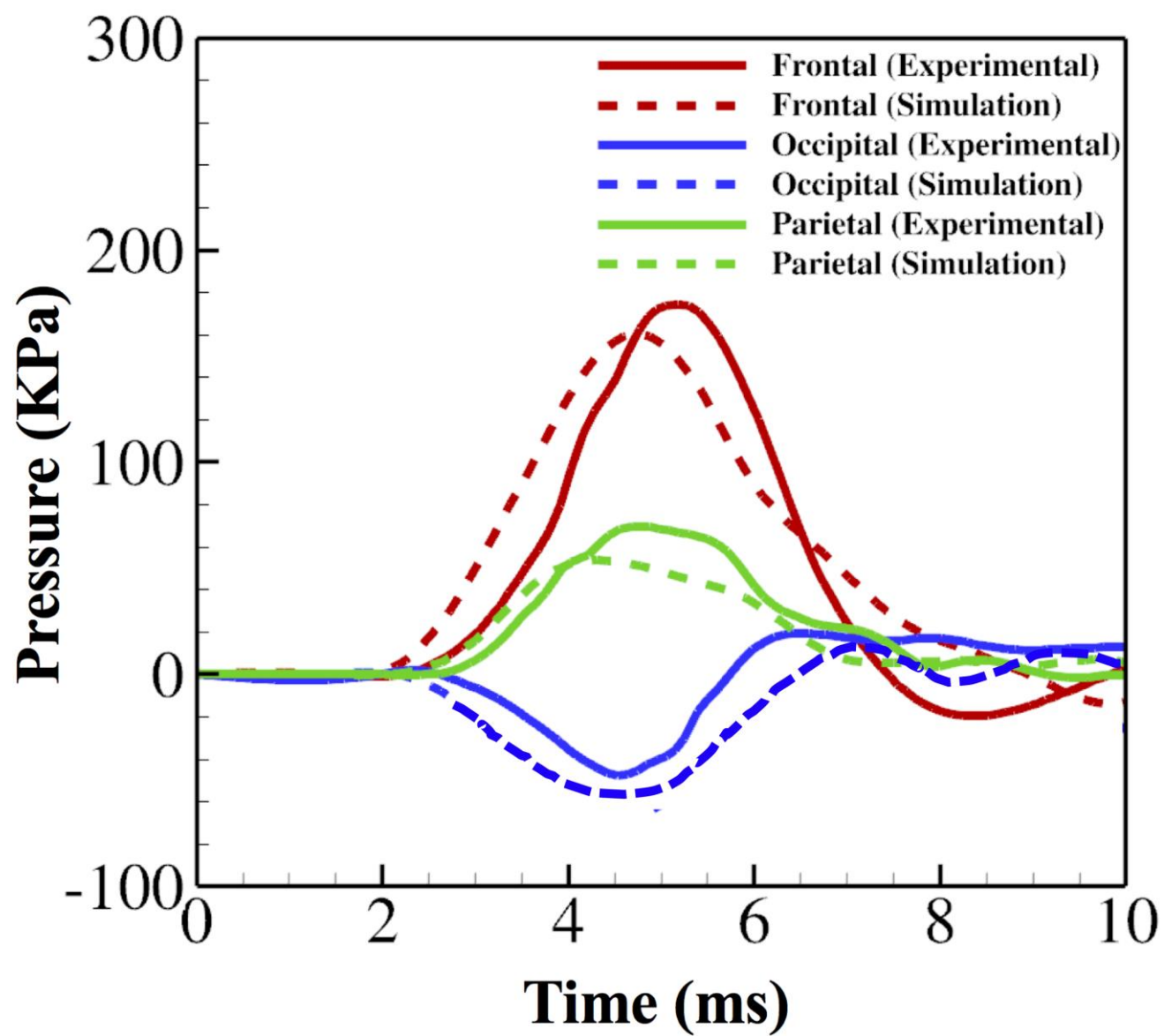

Supplement: S2 Fig — Comparison of experimental and simulated brain-skull relative displacements for frontal loading condition (C383T1)–reported by Hardy et al. [35,36]. (PDF) [file pone.0190881.s004.pdf]

**NDT a1 – X disp.**

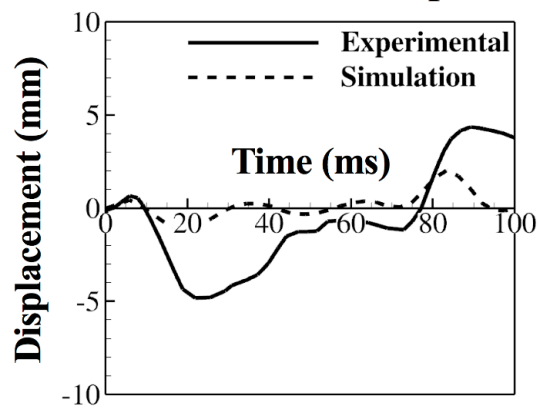

**NDT a1 – Z disp.**

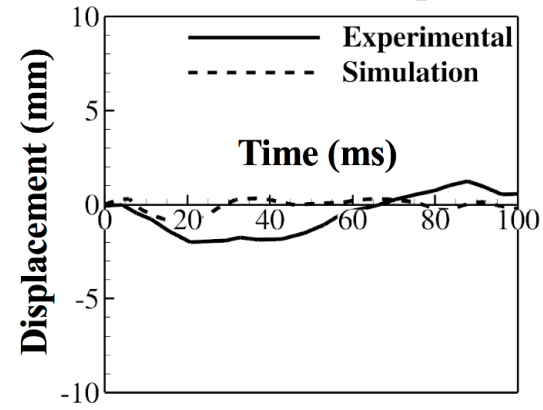

**NDT a6 – X disp.**

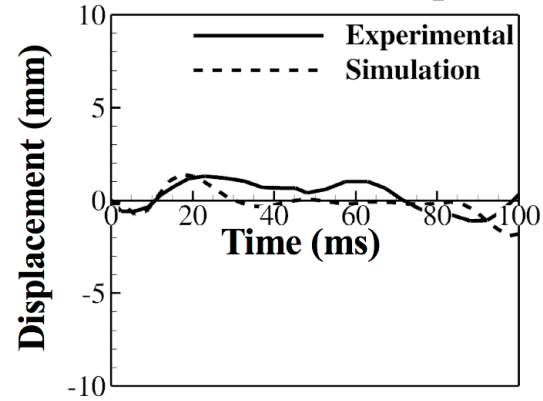

**NDT a6 – Z disp.**

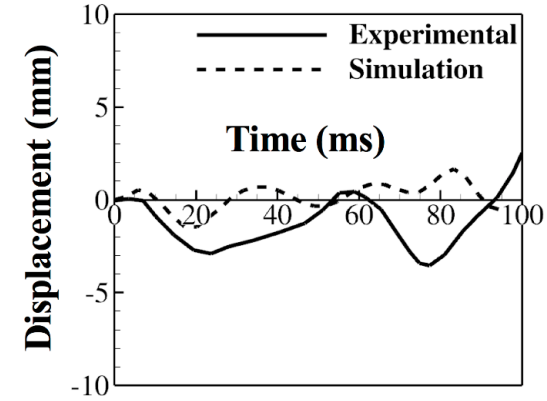

**NDT p1 – X disp.**

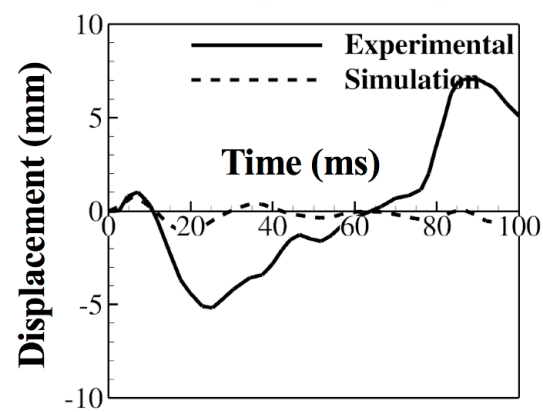

**NDT p1 – Z disp.**

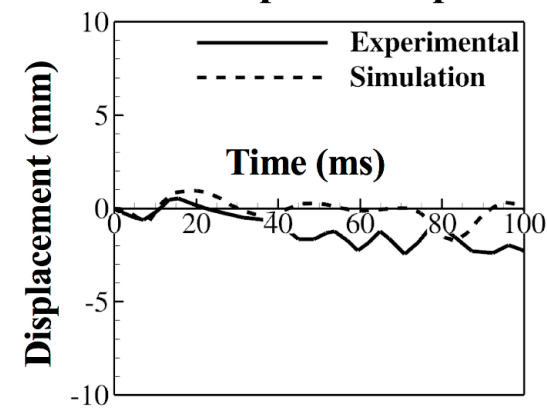

**NDT p6 – X disp.**

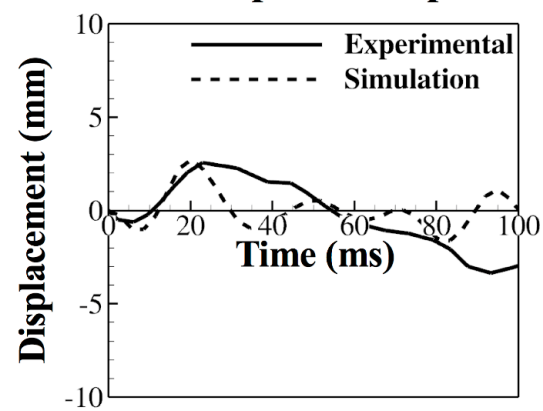

**NDT p6 – Z disp.**

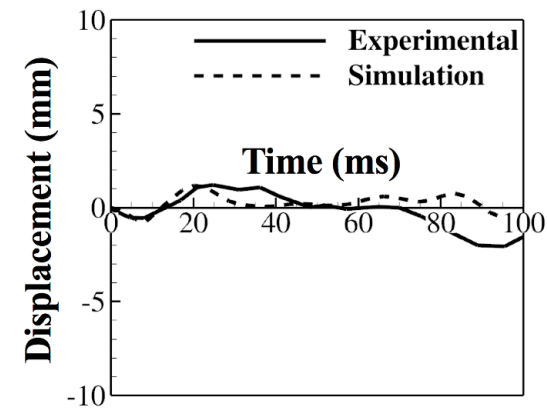

Supplement: S3 Fig — Comparison of experimental and simulated brain-skull relative displacements for occipital loading condition (C755T2)–reported by Hardy et al. [35,36]. (PDF) [file pone.0190881.s005.pdf]

**NDT a1 – X disp.**

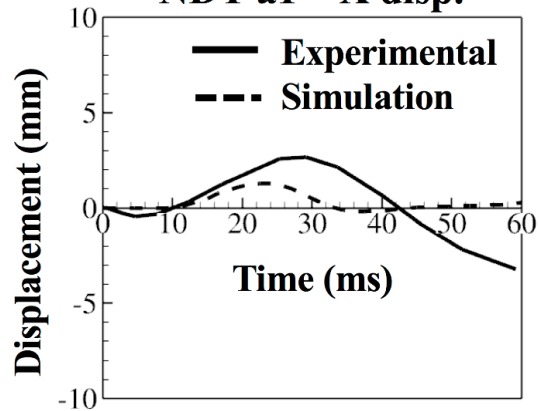

**NDT a1 – Z disp.**

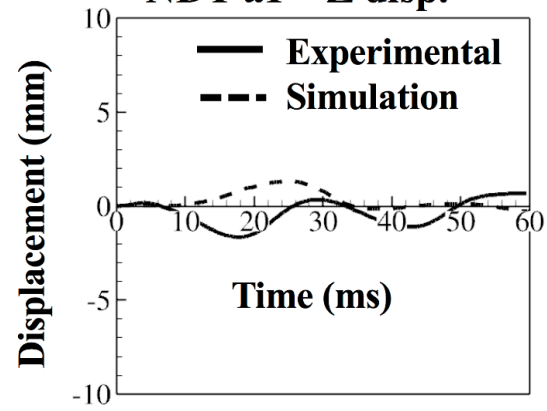

**NDT a6 – X disp.**

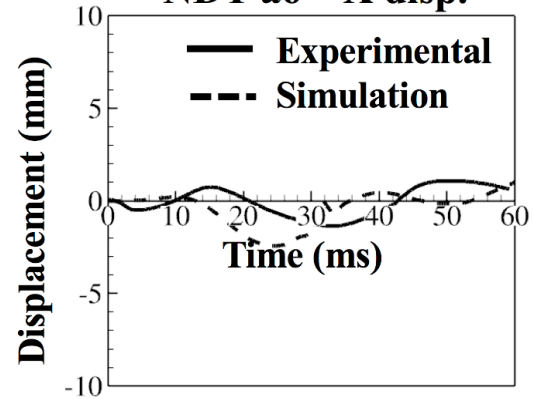

**NDT a6 – Z disp.**

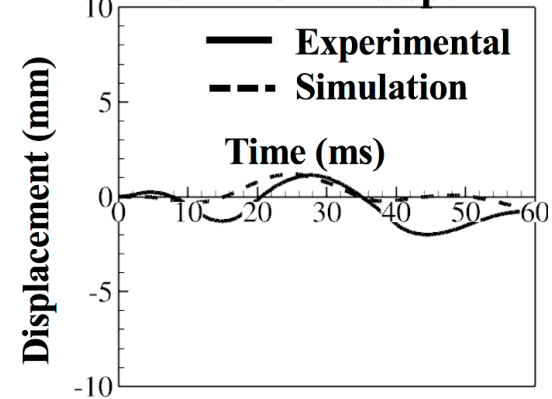

**NDT p1 – X disp.**

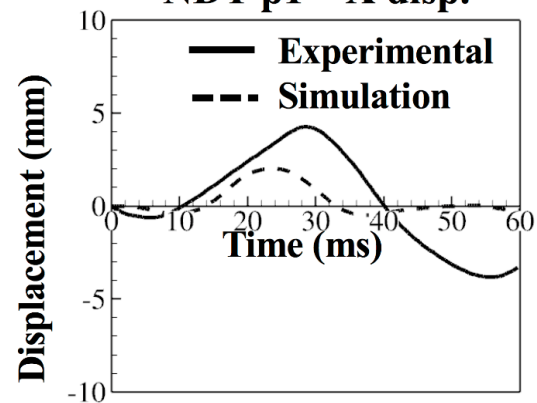

**NDT p1 – Z disp.**

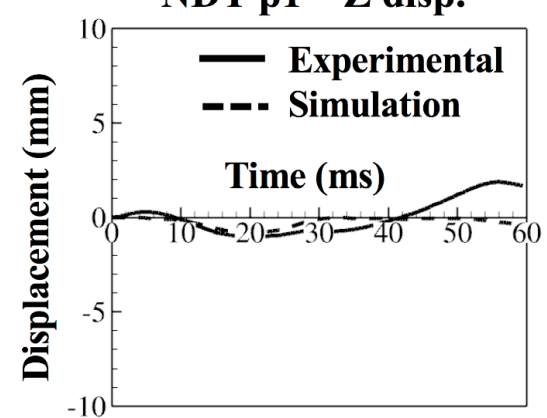

**NDT p6 – X disp.**

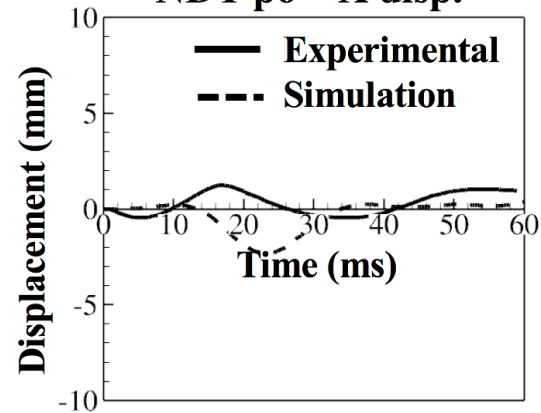

**NDT p6 – Z disp.**

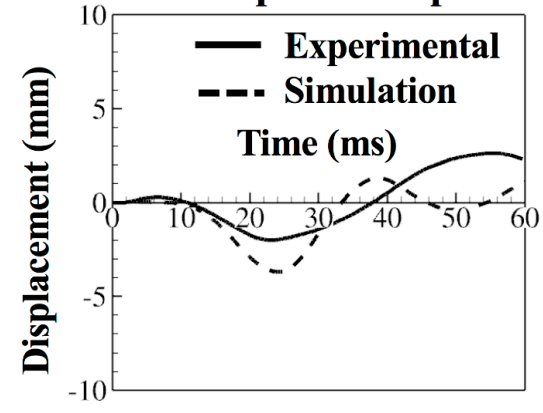

Supplement: S4 Fig — Comparison of experimental and simulated brain-skull relative displacements for parietal loading condition (C393T4)–reported by Hardy et al. [35,36]. (PDF) [file pone.0190881.s006.pdf]

**NDT a4 – Y disp.**

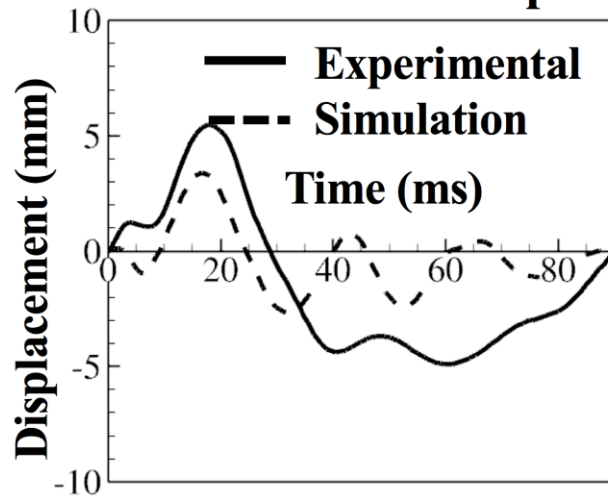

**NDT a4 – Z disp.**

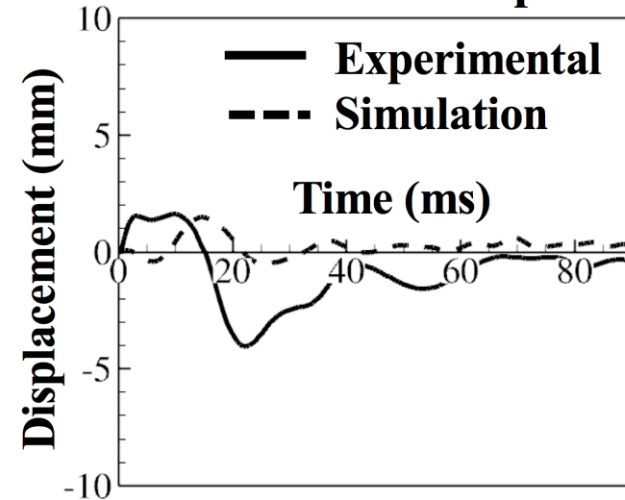

**NDT a11 – Y disp.**

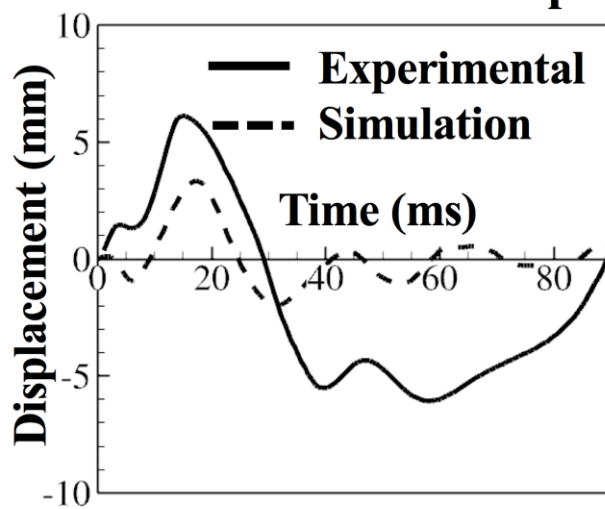

**NDT a11 – Z disp.**

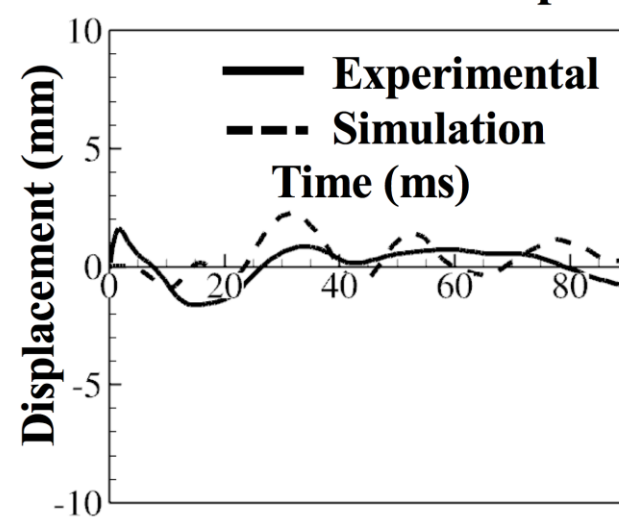

Supplement: S5 Fig — (PDF) [file pone.0190881.s007.pdf]

# Frontal Blast Loading – Female Model

**71 kPa**

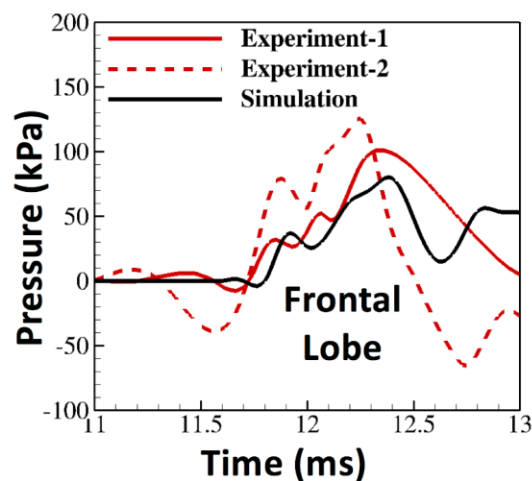

**76 kPa**

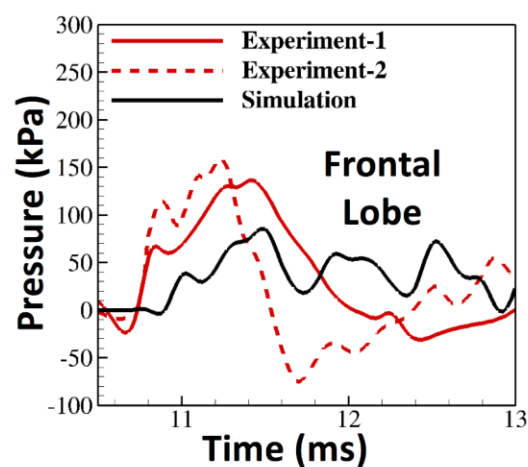

**104 kPa**

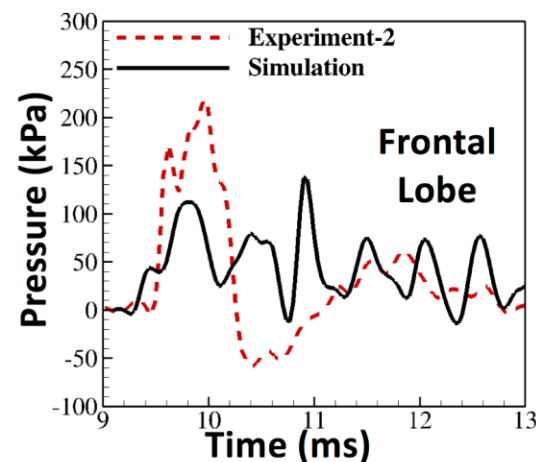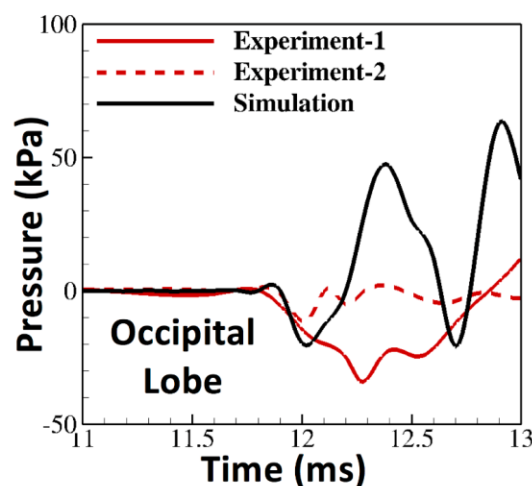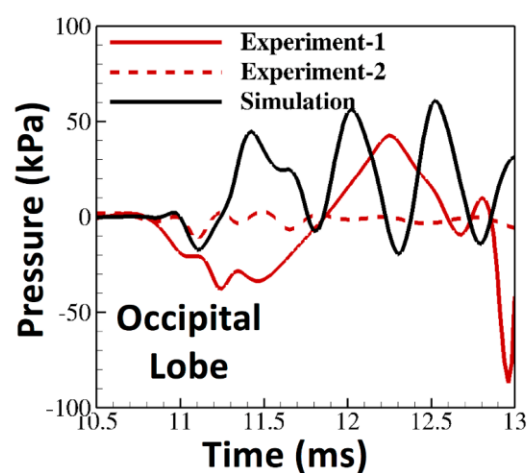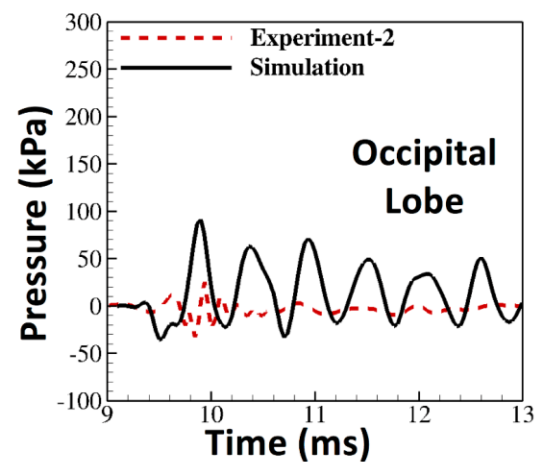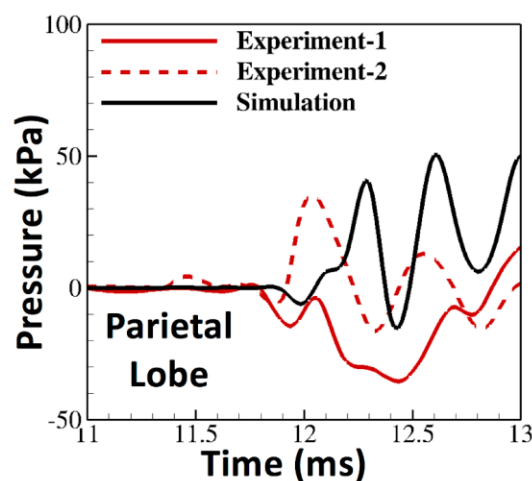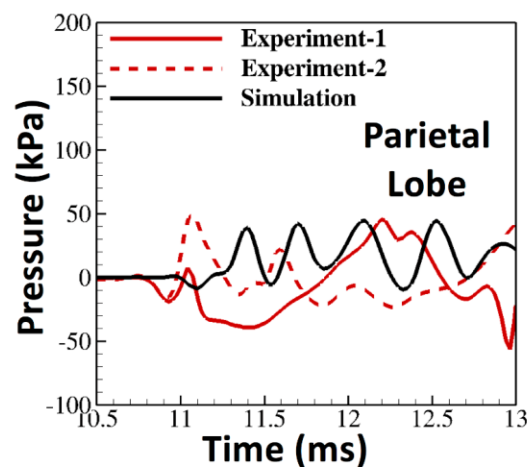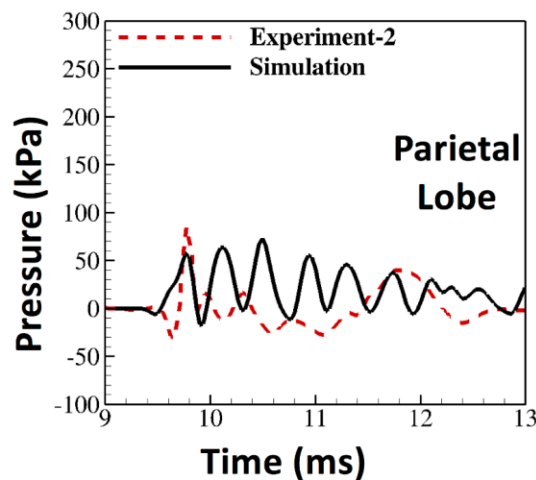

Supplement: S6 Fig — (PDF) [file pone.0190881.s008.pdf]
